# Electrochemical Selective Removal of Oxyanions in a Ferrocene-Doped Metal–Organic Framework

**DOI:** 10.1021/acsnano.4c10206

**Published:** 2024-10-14

**Authors:** Zhi Yi Leong, Jingjing Yao, Niels Boon, Hüseyin Burak Eral, Dong-Sheng Li, Remco Hartkamp, Hui Ying Yang

**Affiliations:** † Pillar of Engineering Product Development (EPD), Singapore University of Technology and Design, 8 Somapah Road, Singapore 487372, Singapore; ‡ Process & Energy Department, Delft University of Technology, Leeghwaterstraat 39, Delft 2628 CB, The Netherlands; § College of Materials and Chemical Engineering, Key Laboratory of Inorganic Nonmetallic Crystalline and Energy Conversion Materials, China Three Gorges University, Yichang 443002, China

**Keywords:** selective ion adsorption, metal−organic framework, electrosorption, water remediation, ferrocene-doped

## Abstract

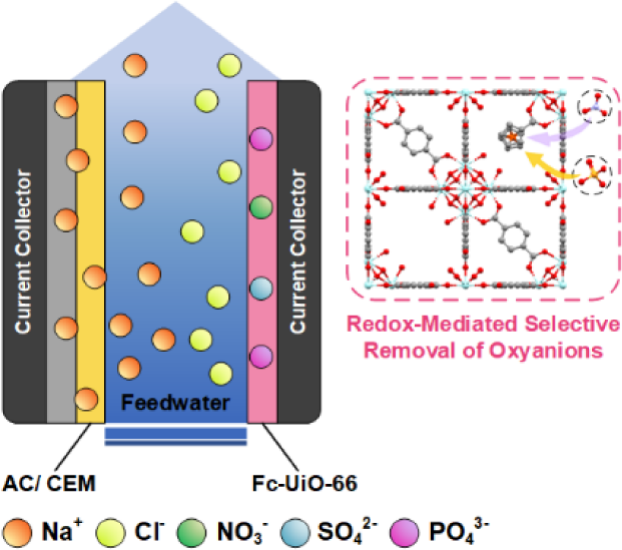

Metal–organic frameworks (MOF) are a class of crystalline, porous materials possessing well-defined channels that have widespread applications across the sustainable landscape. Analogous to zeolites, these materials are well-suited for adsorption processes targeting environmental contaminants. Herein, a zirconium MOF, UiO-66, was functionalized with ferrocene for the selective removal of oxyanion contaminants, specifically NO_3_^–^, SO_4_^2–^, and PO_4_^3–^. Electrochemical oxidation of the embedded ferrocene pendants induces preferential adsorption of these oxyanions, even in the presence of Cl^–^ in a 10-fold excess. Anion selectivity strongly favoring PO_4_^3–^ (*S*_oxy/comp_ = 3.80) was observed following an adsorption trend of PO_4_^3–^ > SO_4_^2–^ > NO_3_^–^ > (10-fold)Cl^–^ in multi-ion solution mixtures. The underlying mechanisms responsible for ion selectivity were elucidated by performing *ex situ* X-ray photoelectron spectroscopy (XPS) on the heterogeneous electrode surface postadsorption and by calculating the electronic structure of various adsorption configurations. It was eventually shown that oxyanion selectivity stemmed from strong ion association with a positively charged pore interior due to the spatial distribution of charge by oxygen constituents. While ferrocenium provided the impetus for ion migration–diffusion, it was the formation of stable complexes with zirconium nodes that ultimately contributed to selective adsorption of oxyanions.

## Introduction

In recent years, access to clean and potable water has been threatened by industrial pollution, climate change, and geopolitical conflict. Agriculture for one is a major contributor to water pollution. Rampant use of chemical fertilizers and mismanagement of environmental assets culminate in agricultural runoff, which, when left unchecked, can result in groundwater contamination.^1,2^ Common contaminants from nutrient-rich runoff include nitrate (NO_3_^–^), sulfate (SO_4_^2–^), and phosphate (PO_4_^3–^) oxyanions. The proliferation of these compounds in the environment can cause widespread ecological damage and pose severe health risks.^3^ Traditional sorbent materials such as zeolites or activated carbon have been deployed as “end-of-pipe” solutions^4^ yet suffer from poor separation efficiency when handling low to trace contaminant concentrations. Separation efficiency is also impacted when other ions or molecules vie for limited adsorption sites. Improving the separation efficiency of sorbent materials entails more precise and discriminated ion adsorption.

Raw sorbent materials can be imbued with ion-selective properties through physiochemical modification methods. Activated carbon, for example, can be pyrolyzed using different chemical reagents to regulate its pore size and, consequently, achieve selectivity through ion-pore size exclusion. More complex methods involve modifying the local environment of binding sites with complexing functional pendants^5,6^ to facilitate highly specific reactions. Among the vast variety of porous materials, reticular crystalline materials such as metal–organic frameworks (MOFs) provide unparalleled customizability through the judicious selection of inorganic metal nodes and organic ligand linkers. A typical MOF such as UiO-66 is constructed from a unit of hexa-zirconium(IV)-oxo, hydroxo, aqua node connected to twelve 1,4-benzene dicarboxylate (BDC) ligands.^7^ UiO-66 possesses high specific surface areas averaging at ∼1200 m^2^ g^–1^, triangular pore windows of ∼6 Å, and high stability over a range of pH values in aqueous solution. Even in an unmodified state, the uniform, rigid pores of UiO-66 provide moderately effective size-exclusion-based separation in gas^8^ and liquid mixtures.^9^ In addition, unsaturated zirconium nodes endow it with highly catalytic Lewis acid sites, which can also double as binding sites to select ions.^10,11^ The extent of binding specificity depends on the type and oxidation state of the metal node in conjunction with the spatial arrangement of ligands.^12^ In some cases, ligands with attached moieties can be aligned in cooperative geometry with nodes to entrap guest species.^13^ This strategy is reminiscent of molecular sensors using porphyrins and anthracene fragments as ion- or molecule-specific cavities.^14^

Research on MOFs for ion separation has been primarily focused on MOFs as adsorbents^11,15−17^ or as membranes.^8,9,18^ Although these approaches prove effective under some conditions (e.g., removal of hexavalent chromium from wastewater),^16^ they cannot be easily adapted to use cases where solution composition is entirely different (e.g., hexavalent chromium from mining deposits). Furthermore, challenging situations arise when a specific ion composition is desired (e.g., dialyzate) or when optimal levels of certain ions need to be maintained (e.g., aquaculture and laboratory tissue culture). Under these conditions, ion composition is dynamic and separation needs to be highly selective, rapidly tunable, and robust; demands that can be fulfilled by electrochemical separation.

Electrochemical separation techniques, particularly those of electrosorption, consist of a pair of electrodes sandwiching a channel containing the feed solution. Electrodes are charged, and ions are removed due to electrostatic attraction. These systems are entirely modular and provide fast ion removal kinetics, and electrodes can be regenerated over multiple charge–discharge cycles. Another advantage that is not widely discussed is the ease at which these electrical systems can be integrated into smart, decentralized networks.^19^ These systems can be implemented as part of water quality monitoring networks in agriculture, manufacturing, or mining industries. On paper, MOFs are ideal electrode materials for ion-selective electrochemical separation, yet MOFs are often bogged by poor electrical conductivity and water stability.^20,21^ To mitigate these shortcomings, MOFs can be carbonized through pyrolysis to obtain MOF-derived carbon products. However, pyrolysis can cause concurrent precipitation of metals and partial collapse of pores.^22^ In addition, pyrolysis destroys the local chemical environment afforded by the nodes and ligands.

**Figure 1:**
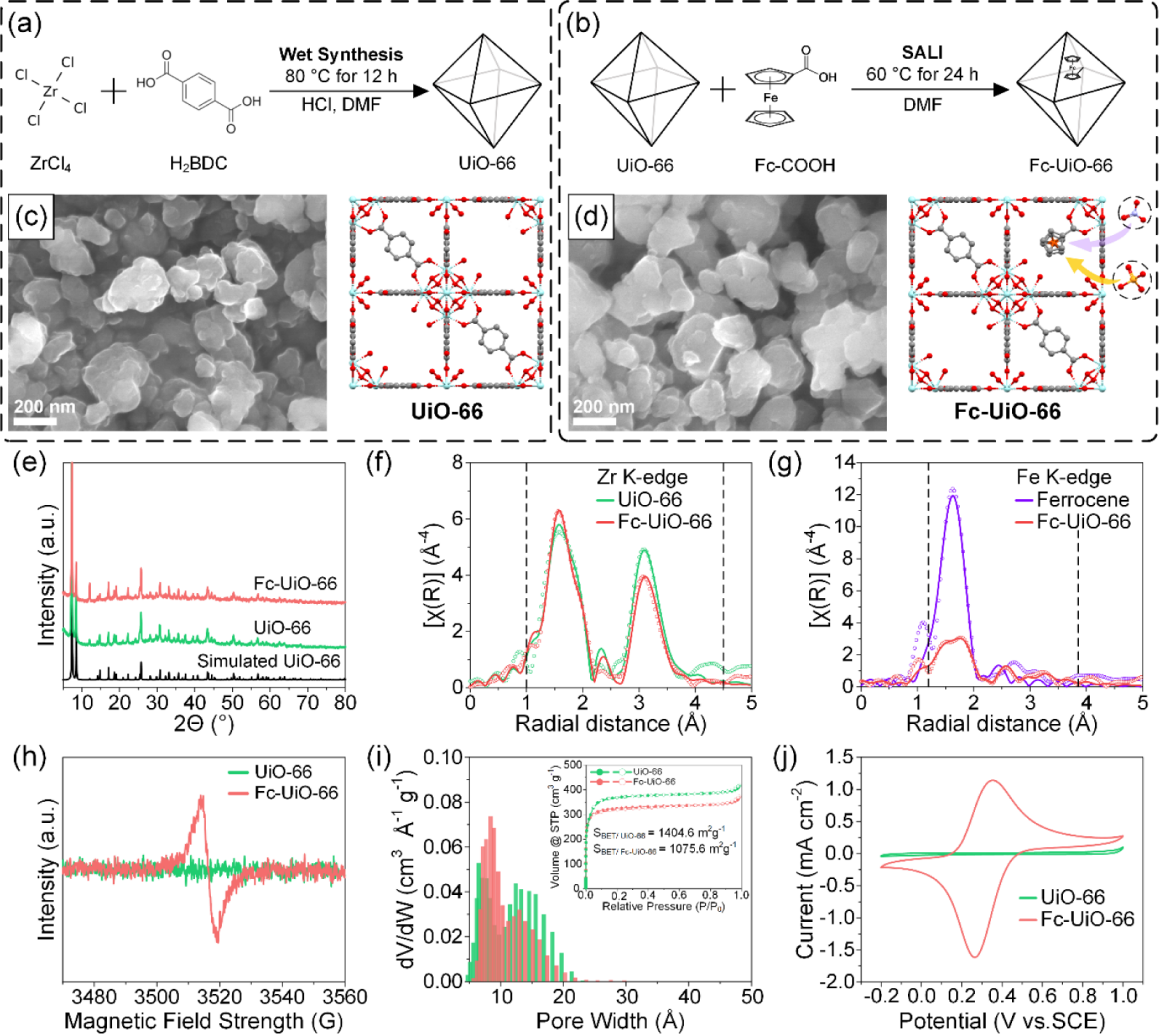
Schematics depicting synthesis protocol for (a) UiO-66 and (b) Fc-UiO-66. SEM images of (c) UiO-66 and a schematic representing UiO-66 with missing linker defects; (d) Fc-UiO-66 and a schematic representing Fc-UiO-66 with a ferrocene pendant. When oxidized to ferrocenium, counterions (e.g., NO_3_^–^ and PO_4_^3–^) engage in competitive adsorption to maintain charge neutrality. (e) PXRD patterns. Phase-uncorrected Fourier transform of *k*^3^-weighted EXAFS data (magnitude) taken at (f) Zr K-edge for UiO-66 and Fc-UiO-66 and (g) Fe K-edge for pure ferrocene and Fc-UiO-66. (h) EPR spectra. (i) Pore size distribution with inset showing N_2_ adsorption–desorption isotherms. (j) CV curves obtained in a 0.1 M NaCl solution.

In this work, we demonstrate the use of ferrocene-doped UiO-66 as a water stable and redox-active MOF for the selective electrochemical removal of oxyanions. Under normal circumstances, UiO-66 is poorly conductive (∼10^–7^ S cm^–1^)^23^ and exhibits negligible charge. To compensate for poor electrical conductivity, ferrocene pendants were introduced through a postsynthesis solvent-assisted ligand incorporation (SALI) process^24^ (Figure 1b). Studies have shown that charge transport can be stimulated through consecutive discrete charge hopping events across redox-active components such as redox-active ligands, metallocene pendants,^25^ and open metal sites.^26,27^ Reducing or oxidizing the redox-active component displaces local charge equilibrium and compels mass transfer of corresponding counterions to maintain charge neutrality (Figure 1d). The diffusion of the charge balancing ion is affected by (1) ion concentration, size, and charge, (2) pore dimensions of MOF, (3) charge environment of pore interior provided by the node and ligand arrangement. These factors provide fertile ground for selective manipulation of ion transport. In the case of oxyanions, our experiments showed preferential removal of ions following PO_4_^3–^ > SO_4_^2–^ > NO_3_^–^ (>Cl^–^ in 10-fold background concentration) in multi-ion mixtures. The difference in ion removal was mainly due to ion association with positive charges of the pore interior and not due to steric hindrance, as one would expect. *Ex situ* X-ray photoelectron spectroscopy (XPS) on electrode postadsorption and density functional theory (DFT) simulations suggested an adsorption mechanism heavily regulated by metal coordination with either ferrocenium iron or UiO-66 zirconium with stronger contribution from the latter. Our findings will have broader implications for MOF materials in environmental remediation beyond adsorbent or membrane-type applications, and other water-stable MOFs can also be similarly adapted for aqueous electrochemical separation.

## Results

### Structural Characterization of UiO-66 and Fc-UiO-66

Precise determination of atomic positions and composition was accomplished through powder X-ray diffraction (PXRD) and extended X-ray absorption fine structure (EXAFS) data analysis. A cursory comparison between the diffraction patterns of pristine UiO-66 and Fc-UiO-66 with a simulated diffraction pattern (COD #4132636)^28^ showed that no major differences save for an increase in peak intensity at 12° corresponding to the (220) plane for Fc-UiO-66 (Figure 1e). This is likely due to the occupancy of leftover solvent molecules and/or ferrocene pendants. Overall, the similarity in diffraction patterns indicates that the crystalline structure was largely preserved after ferrocene doping. Detailed analysis was further performed using Rietveld refinement, and the structure solution of UiO-66 was solved in the *Fm**m* space group (no. 225). A reasonably high degree of fit was obtained (*R*_wp_ = 0.0778, *R*_exp_ = 0.0508, and χ^2^ = 2.335, Table S1), and stoichiometric composition was determined to be Zr_6_C_39.4_H_23.3_O_27.7_, which roughly translates to one missing BDC linker per Zr cluster, a result well supported by thermogravimetric analysis (Figure S5). The loss of a linker leaves the zirconium node vulnerable to functionalization^24,29^ via a carboxylate tether from the ferrocene derivative.

Successful doping of ferrocene was confirmed through electron paramagnetic resonance (EPR) spectroscopy and quantified by using inductively coupled plasma (ICP) spectroscopy. EPR spectra of the as-synthesized Fc-UiO-66 (Figure 1h) show a single pair of asymmetric peaks belonging to iron in contrast to the relatively unremarkable spectra of UiO-66. Ferrocene content was measured using ICP and amounted to approximately 1.24 units per Zr cluster. Understanding the local environment of ferrocene is crucial to unraveling the mechanism behind redox-mediated counterion transfer and adsorption. EXAFS spectra at Zr K-edge and Fe K-edge of Fc-UiO-66 were analyzed with respect to UiO-66 and pure ferrocene, respectively. Figure 1f shows a comparison between the *k*^3^-weighted Fourier transformed EXAFS data of UiO-66 and Fc-UiO-66 at the Zr K-edge. Two prominent features were detected at 1.6 and 3.1 Å with little deviation in radial distance. Using crystal data from the refined UiO-66 structure, first shell scattering was attributed to single scattering paths Zr–O_μ3_ and Zr–O, whereas second shell scattering was largely due to backscattering from closest coordinated Zr neighbors. Spectra of Fc-UiO-66 notably differed from those of UiO-66 in two ways: sharper and more intense features at 1.6 Å and a decrease in peak intensity at 3.1 Å. Sharp features at 1.6 Å indicated increased ligand coordination due to functionalization with ferrocenecarboxylic acid, while a decreased intensity at 3.1 Å was consistent with loss of coordinated Zr neighbors (from CN_Zr_ 4.17 to 3.33). An average loss of Zr neighbors could be, in part, due to elongation of the Zr–O bond tethered to ferrocene.

Analysis of EXAFS data at the Fe K-edge provided much insight into the structural differences between pure ferrocene and ferrocene in UiO-66 (Figure 1g). First shell scattering of Fe in pure ferrocene was fitted to Fe–C single scattering paths corresponding to cyclopentadienyl carbons with secondary contribution from a multiple scattering path originating from two adjacent carbons. The average Fe–C bond distance was determined to be 2.063 Å, which is consistent with literature data.^30,31^ In contrast, the spectral features of ferrocene in UiO-66 were more complex and far less intense. The broad feature between 1.2 and 2.2 Å was associated with two Fe–C scattering paths corresponding to effective radii of 2 and 2.1 Å, while the sharp feature at 2.6 Å was due to carbon scatterers situated on an outstretched region of the cyclopentadienyl ring. The distortion of the ferrocene geometry was likely brought about by weakening of the ligand field^32^ due to carboxylate tether with a Zr node. This argument is also justified by the presence of an EPR signal in otherwise low-spin Fe(II) complexes, such as ferrocene. A smaller, broad feature at 3.4 Å was associated with carbon in the described carboxylate tether. Further details on EXAFS fitting and parametrization are described in Note S2.

The occupancy of ferrocene, like most other guest species, will inevitably decrease the specific pore volume and surface area. The surface area of UiO-66 in particular decreased by about 23% after ferrocene doping, whereas its total pore volume suffered a loss of about 13%. While the isotherms of both samples were predominantly microporous (Figure 1i inset), there were significant differences in pore size. The pore size distribution of UiO-66 in Figure 1i shows a sharp feature culminating at approximately 6.07 Å, accompanied by a broad feature between 12 and 23 Å. A primary pore width of 6.07 Å is consistent with aperture sizes of UiO-66, while the broad feature suggests a distribution of pore sizes enlarged by missing linker defects. Fc-UiO-66 on the other hand showed a slight increase in primary pore width (8.04 Å), while the accompanying broad feature became significantly less intense. When considered alongside structural evidence from PXRD and EXAFS, we hypothesized that the inclusion of ferrocene had partially filled in the voids left from missing linker defects.

### Electrochemical Properties of Fc-UiO-66

Cyclic voltammetry (CV) curves of the MOF samples are displayed in Figure 1j. The CV curve of Fc-UiO-66 distinctively showed a pair of redox peaks centered around 0.31 V, whereas no discernible electrochemical activity was observed for UiO-66. The highly symmetric peaks indicate a highly reversible redox process not bogged by poor electrical conductivity or limited counterion diffusion. This contrasts with previous work by Palmer et al.^24^ where the CV curve for a ferrocene-doped sample of UiO-66 showed highly asymmetric redox behavior and an arduous oxidation process. We ascribe the differences in electrochemical response to different electrode fabrication techniques (slurry coating vs film deposition), where the use of conductive multiwalled carbon nanotubes (MWCNTs) enhanced electron transport. The capacitive influence of MWCNTs if any would have manifested as semirectangular features in the CV curve, yet no such features appear. To quantify the percentage of electroactive ferrocene, Fc-UiO-66 electrodes were completely oxidized by applying 0.4–1 V for 15 min in a 0.1 M NaCl solution. The total charge transferred during oxidation was calculated and compared to the total amount of ferrocene present in the electrode. Figure S6 shows an increase from 39% to 55% of utilized ferrocene as the oxidation potential steadily ramped up. A portion of the embedded ferrocene remained inaccessible due to progressively slower counterion diffusion in the internal structure, limited self-exchange reactions, or ion overcrowding in pores.

**Figure 2:**
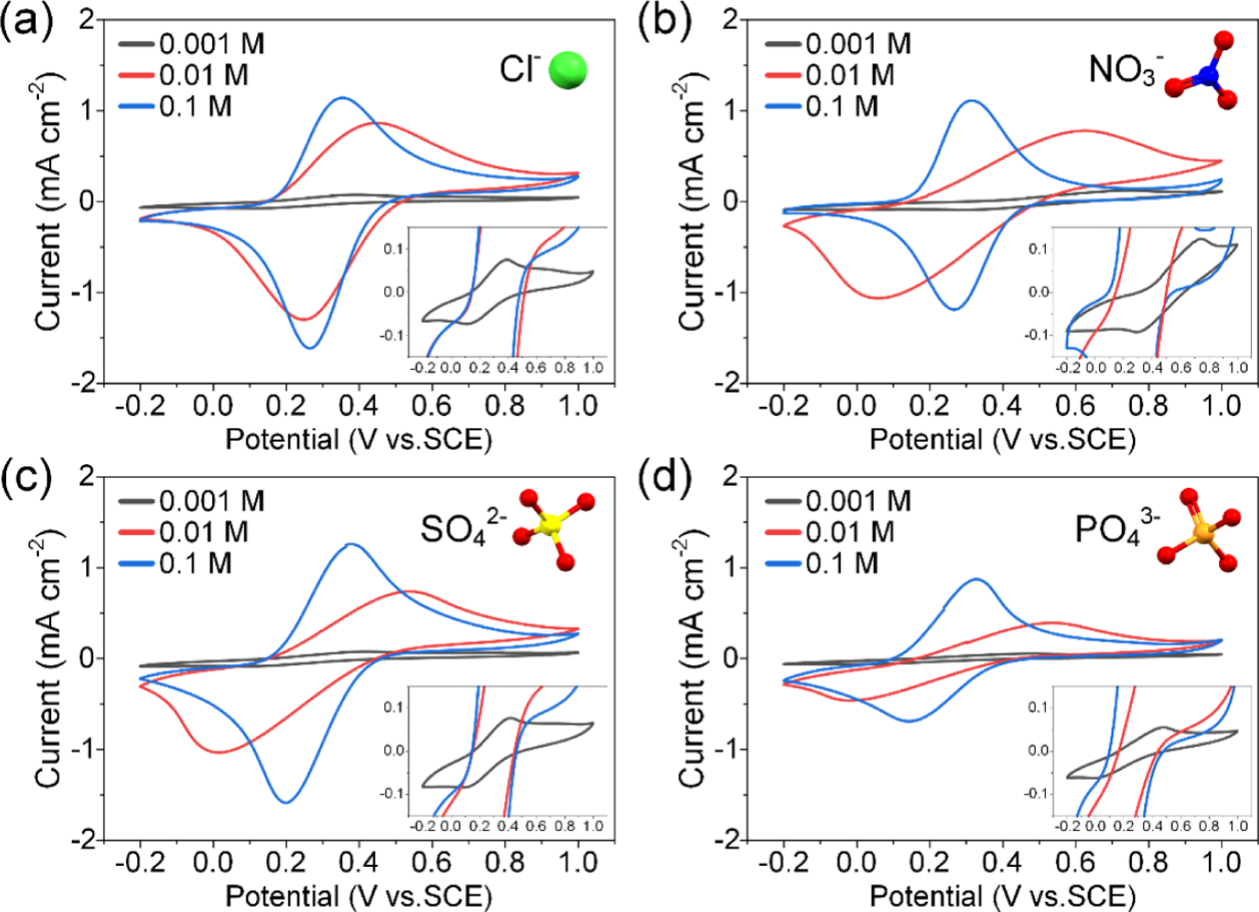
CV curves of Fc-UiO-66 in concentrations of 0.001, 0.01, and 0.1 M for (a) NaCl, (b) NaNO_3_, (c) Na_2_SO_4_, and (d) NaH_2_PO_4_. Experiments were performed using a 3-electrode setup by applying a scan voltage of 5 mV s^–1^ across a voltage window of −0.2 to 1.0 V. Insets show close-up of the CV curve in a 0.001 M salt solution.

The redox activity of ferrocene in Fc-UiO-66 was further investigated in salt solutions representing oxyanions of interest along with NaCl acting as a reference (Figure 2). Cl^–^ was used as a model anion due to its small size and ease of infiltration. At 0.1 M, the CV curve of Cl^–^ showed a typical pair of symmetric redox peaks centered at *E*_1/2_ = 0.31 V. When the concentration was decreased by a factor of 10, oxidative and reductive peaks started to broaden and shift. A further 10-fold reduction in the concentration resulted in severely diminished current densities and a heavily distorted voltammogram. At high Cl^–^ concentration, diffusion was fast enough to counterbalance the positively charged ferrocenium on the surface and within MOF crystallites.^25,26^ However, as the anion concentration decreased, bulk diffusion slowed, and ferrocene embedded deep within the MOF cannot be accessed efficiently. These observations were also apparent across NO_3_^–^, SO_4_^2–^, and PO_4_^3–^, yet there appeared to be other factors at play. While voltammogram distortion was visible only at 0.001 M for Cl^–^, it became visible at 0.01 M for oxyanion solutions. At 0.01 M, shifts in oxidative and reductive peaks had caused voltammogram shearing, and *E*_1/2_ shifted to lower potentials. This could be caused by steric hindrance and pore blockage since oxyanions were larger and bulkier. However, N_2_ sorption results showed the existence of sufficiently large pores (pore width: ∼8.04 Å; hydrated radii of Cl^–^: 3.32 Å, NO_3_^–^: 3.35 Å, SO_4_^2–^: 3.79 Å, and PO_4_^3–^: 3.39 Å).^33,34^ If size effects were apparent, then CV curves should exhibit much lower current densities (especially between Cl^–^ and SO_4_^2–^). Instead, similar current densities were observed, which indicated that an equivalent amount of ferrocene/ferrocenium was accessed. Hence, the inefficiency in ion transport was likely caused by ion association with ferrocenium within the confined space of the pore interior. The ease at which an anion is adsorbed or released directly influences the rate at which the redox reaction proceeds. In the case of the highly charged trivalent PO_4_^3–^, it is strongly associated with the positively charged pore environment of both the Zr node and ferrocenium. This hampers diffusion and results in a lower current density.

### Electrochemical Redox-Mediated Anion Adsorption

**Figure 3:**
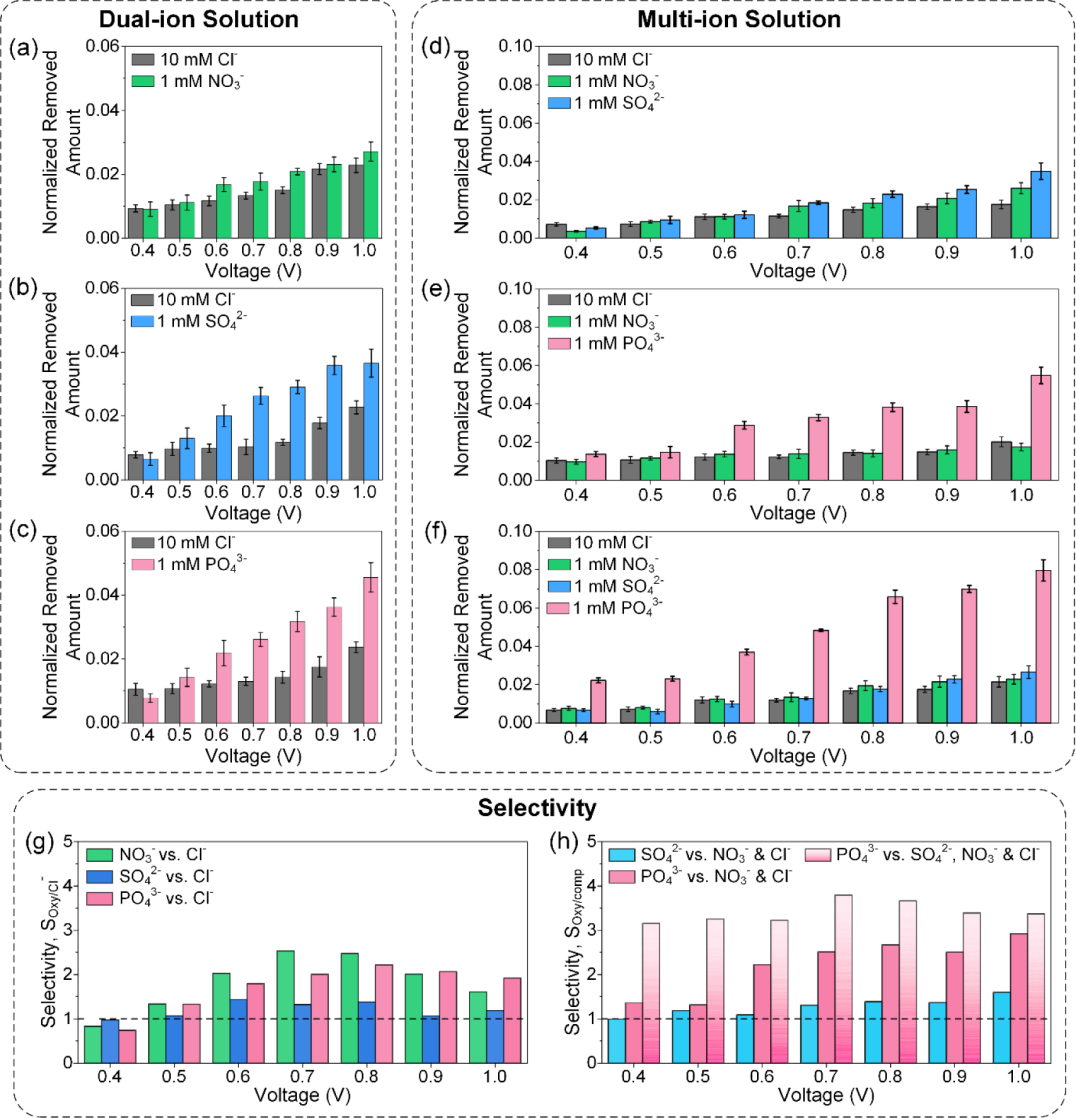
(a–c) Normalized redox-mediated adsorption in dual-ion solutions of 1 mM (a) NO_3_^–^, (b) SO_4_^2–^, and (c) PO_4_^3–^ with 10 mM Cl^–^ as background. (d–f) Normalized redox-mediated adsorption in multi-ion solutions of 1 mM (d) NO_3_^–^ and SO_4_^2–^; (e) NO_3_^–^ and PO_4_^3–^; and (f) NO_3_^–^, SO_4_^2–^, and PO_4_^3–^ with 10 mM Cl^–^ as background. Selectivities of oxyanions in (g) dual-ion solutions and (h) multi-ion solutions. An ion-selectivity of 1 (dashed line) indicates no selectivity.

Redox-mediated adsorption of oxyanions was investigated via a series of experiments employing dual-ion and multi-ion solution mixtures. The dual-ion solution mixture consisted of 1 mM sodium-based oxyanion salt along with 10 mM NaCl as the background. To adsorb the ions, the Fc-UiO-66 anode was oxidized at constant potentials of 0.4–1 V for 15 min with an activated carbon/cation-exchange membrane serving as the cathode. To regenerate the electrodes, Fc-UiO-66 was reduced at −0.2 V for 15 min. The experimental layout is depicted in Figure S7. The normalized amount of ions removed at equilibrium is shown in Figure 3a–c, and preferential removal of oxyanions was observed across all experiments. A notable feature is the lack of selectivity at 0.4 V, which is attributed to incomplete conversion of ferrocene to ferrocenium. At potentials above 0.4 V, all accessible ferrocene would have been converted to ferrocenium, and oxyanion uptake is understandably higher. Within the limits of experimental error, the amount of Cl^–^ adsorbed is fairly consistent across all potentials in all solution mixtures. Oxyanion adsorption when competing against a 10-fold background of Cl^–^ generally follows a trend of PO_4_^3–^ > SO_4_^2–^ > NO_3_^–^ although the calculated selectivities indicated a greater preference for SO_4_^2–^ between the potentials of 0.6 and 0.8 V. The highest ion selectivities were recorded at 2.53 for SO_4_^2–^, 2.21 for PO_4_^3–^, and 1.44 for NO_3_^–^.

The origin of enhanced oxyanion adsorption can be deduced by considering the adsorption of NO_3_^–^ and Cl^–^ as depicted in Figure 3a. Since both NO_3_^–^ and Cl^–^ possessed similar hydrated radii and hydration enthalpies,^35,36^ a higher adsorption of Cl^–^ was expected given the 10-fold concentration advantage. Yet experimental evidence depicted a marginally higher adsorption of NO_3_^–^ over Cl^–^ across oxidation potentials of 0.5–1.0 V, yielding an average selectivity of ∼1.3. This result was attributed to favorable attractive interactions between oxygen constituents of nitrate and the positively charged environment of ferrocenium and zirconium. The effects of ion association manifested more strongly in species carrying a more negative charge, such as SO_4_^2–^ and PO_4_^3–^. While strong ion association can promote ion competitiveness and displace weakly associated Cl^–^, it can also result in slower charge transport and retarded regeneration of the electrode. Cyclability of the anode was investigated using a mixture of 1 mM PO_4_^3–^ with 10 mM Cl^–^ (Figure S8), and ∼97% reversible working capacity was observed during the first cycle with a decay to ∼93% at the end of the 12th cycle. These results strongly suggest that the strength of the ion association was still within the range of reversibility.

To investigate the competitive effects of multiple oxyanions, multi-ion solution mixtures were used, and the adsorption of each ionic component was summarized in Figure 3d–f. The concentration of oxyanions was set at 1 mM, and a background of 10 mM Cl^–^ was used. In previous experiments involving only one type of oxyanion and a background of Cl^–^, the selectivity of SO_4_^2–^ was apparently higher than that of PO_4_^3–^. However, a multi-ion solution mixture directly pitting the two ions against each other proved otherwise. Figure 3f shows that the adsorption of PO_4_^3–^ was distinctly higher across all applied potentials and an apparent selectivity of 3.80 was reached at 0.7 V. In the absence of PO_4_^3–^, SO_4_^2–^ was preferentially adsorbed but to a lesser degree. The apparent selectivity of SO_4_^2–^ hovered at an average of ∼1.4, which was considerably less than what was achieved in the dual-ion solution mixture. The adsorption of NO_3_^–^ in all mixtures mimicked its performance in the dual-ion solution experiment and was only slightly more adsorbed compared to the Cl^–^ background.

The stability and performance of the Fc-UiO-66 anode were further investigated by considering the effects of electrolyte pH between 4 and 10 (Figure S9). There appeared to be no significant change in adsorbed amounts at low pH, yet decreasing adsorption could be observed as the pH was increased. Loss of selectivity set in as early as pH 7.6 for NO_3_^–^ and at pH 8.5 for SO_4_^2–^. While this could be due to increasing competition from hydroxide anions, a more compelling argument was the gradual degradation of the MOF framework due to the increasing linker solubility. Studies have shown that terephthalate linkers of UiO-66 start to leach as pH is increased.^37−39^ The leaching of terephthalate linkers is intimately linked to its p*K*_a_ value (∼3.51 at 298 K), where linker dissociation is expected at pH values above 5,^37^ although various articles report stability up to pH 9.5. The dissociation of linkers destabilizes pore structure and can lead to pore collapse. However, this has less impact on the adsorption of PO_4_^3–^ since PO_4_^3–^ can directly coordinate with the zirconium node and inadvertently help stabilize the pore structure. We note that adsorption/regeneration of PO_4_^3–^ remained reversible for at least 10 more cycles albeit at diminished performances.

### *Ex Situ* XPS Characterization of Electrode Postadsorption

The surface chemistries of as-synthesized, desolvated UiO-66 and Fc-UiO-66 were investigated via XPS, and results are reported under Note S3 along with Figure S10. Elemental quantification based on XPS showed a doping of 3.93 ferrocene units per Zr cluster, contrasting with the bulk value of 1.24. The higher value was likely due to more extensive functionalization on the exposed surface of MOF crystallites. Since a higher density of ferrocene resided on the MOF surface, it stands to reason that counterion transport and adsorption would be most prevalent on the surface.

**Figure 4:**
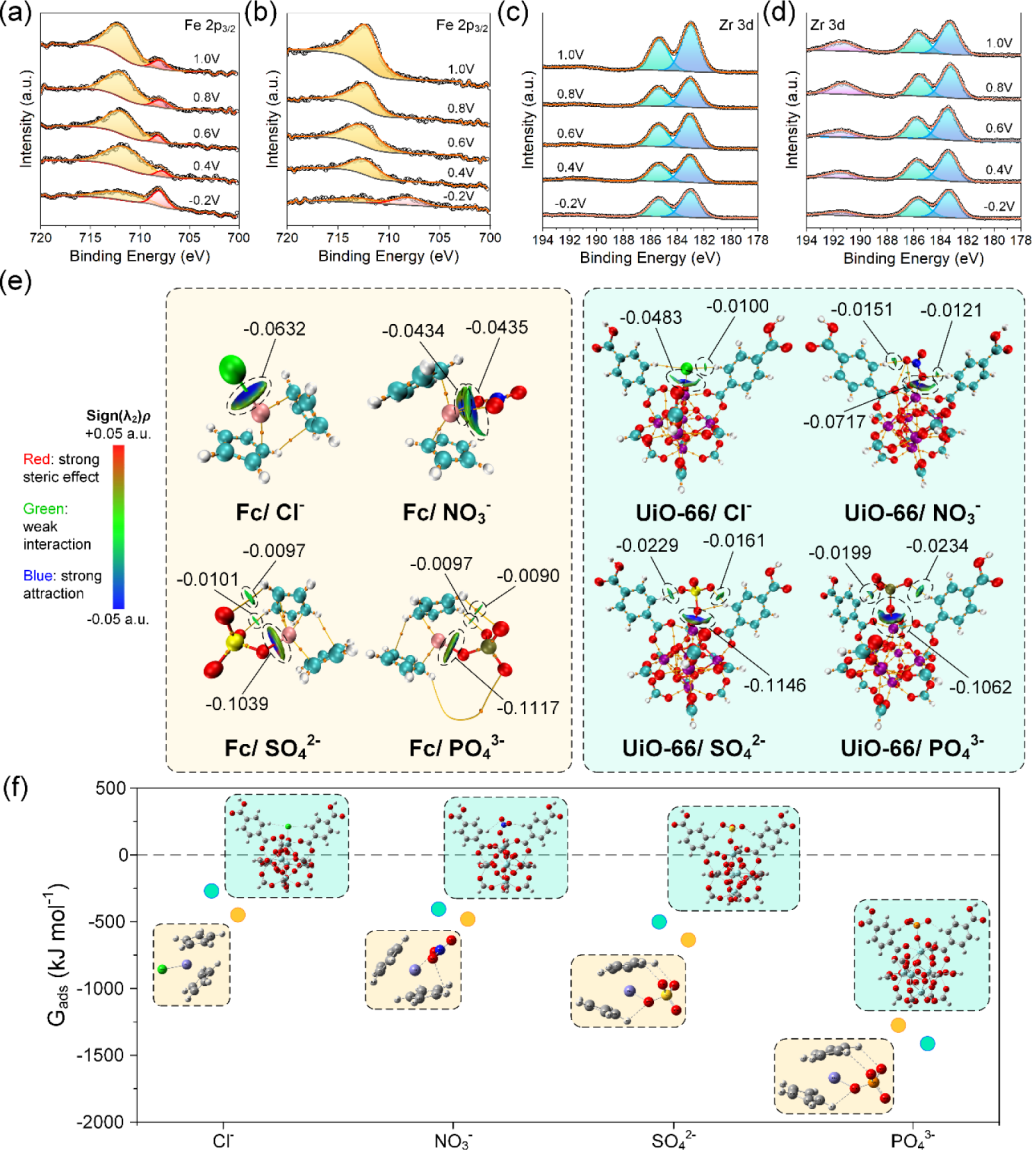
Fe 2p_3/2_ spectra of electrochemically oxidized Fc-UiO-66 anode in (a) 10 mM NaCl solution versus (b) 1 mM NaH_2_PO_4_ and 10 mM NaCl solution. Zr 3d spectra of electrochemically oxidized Fc-UiO-66 anode in (c) 10 mM NaCl solution versus in (d) 1 mM NaH_2_PO_4_ and 10 mM NaCl solution. (e) IGMH–AIM analyses of Cl^–^, NO_3_^–^, SO_4_^2–^, and PO_4_^3–^ association with ferrocenium and UiO-66. (f) Free energy of adsorption (*G*_ads_) for anion associated ferrocenium and UiO-66 associated systems with the corresponding adsorption configurations.

High-resolution *ex situ* XPS spectra of phosphate- and chloride-adsorbed Fc-UiO-66 anodes presented in Figure 4a–d show Fe 2p_3/2_ and Zr 3d regions. The Fe 2p_3/2_ region is mainly comprised of two component peaks located at ∼711 eV and ∼708 eV, corresponding to Fe^3+^ and Fe^2+^ states, respectively. The relative area ratio of the two component peaks was used to track redox changes, and an increase in area ratio of Fe^3+^ to Fe^2+^ could be observed when oxidation potential was raised from −0.2 to 1.0 V. Ferrocenium had begun to form at >0.4 V and by 0.8 V, all accessible ferrocene was oxidized to ferrocenium. In the case of PO_4_^3–^, the Fe^3+^ component far surpassed the Fe^2+^ component and virtually no Fe^2+^ component was detected after 0.4 V. The Fe^3+^ component peak was also slightly shifted to ∼712.4 eV, which indicated stronger interaction between Fe and PO_4_^3–^. The Zr 3d region exhibited two peaks, which are characteristic of UiO-66 compounds in literature,^40−42^ save for the emergence of a broad feature (fwhm = 2.20–2.36 eV) centered at 191.4 eV when PO_4_^3–^ was engaged. Using Zr 3d_5/2_ as a reference, an increase in relative intensity was observed as the oxidation potential increased, evidently suggesting a redox-mediated interaction between Zr and PO_4_^3–^.^43^ The relative area ratio between the broad feature and Zr 3d_5/2_ was calculated and plotted in Figure S12, showing a value of 0.2 when the anode was reduced and 0.48 when oxidized to 0.8 V.

### DFT Calculations of Anion Adsorption

The nature of ion adsorption and association was further explored using DFT. To properly evaluate intermolecular interactions, the electronic structures (HOMO, LUMO, *E*_gap_, ESP, and LOL) of all anions, ferrocenium, and UiO-66 were first determined and described in Figures S13–S15 and Tables S5 and S7. The effects of solvation were considered under SMD, and anion adsorption was independently addressed in UiO-66 or ferrocenium associated systems. Qualitatively speaking, this is a reasonable approximation since the closest distance between a UiO-66 node and a ferrocenium pendant is more than 3.5 Å, and adsorption effects are prominent only within 3.5 Å. In our models, we neglect overlapping effects and consider only the influence of a UiO-66 node or a ferrocenium pendant. Optimized adsorption configurations of ferrocenium associated and UiO-66 associated systems are presented in Figure S16 with the relevant bonding parameters listed under Tables S7 and S8.

A general distortion in ferrocenium geometry was observed in the formation of anion–ferrocenium complexes with direct participation from ferrocenium iron. While chloride directly interacted with iron, oxyanions adopted more complex configurations, with constituent oxygen atoms actively engaging ferrocenium iron and cyclopentadienyl hydrogens cooperatively. The interatomic distance between iron and a nearby constituent oxygen was calculated to be 2.244, 1.883, and 1.857 Å for NO_3_^–^, SO_4_^2–^, and PO_4_^3–^, respectively. These results seemed to oppose the conventional wisdom that larger anions would experience higher degrees of steric hindrance from cyclopentadienyl rings, which would consequently limit their interactions with iron. The effects of electrostatic attraction if any would be minimized due to delocalization of positive charge by the cyclopentadienyl rings.^44,45^ An analysis of electron density maps revealed elevated electron localization along cyclopentadienyl rings corresponding to specific oxygen–hydrogen interactions (Figure S17) spanning distances of ∼2.5–2.9 Å. It was likely that the positive charge gained through oxidation was redistributed across the cyclopentadienyl rings, which induced an attractive interaction with oxygen constituents on the oxyanion. This caused further charge delocalization between the rings and iron, which in turn, reduced the covalency between them and enabled the formation of a metal coordination bond between iron and another constituent oxygen. In the case of oxyanion-UiO-66 associated systems, a constituent oxygen was shown to engage in bond formation with zirconium, while other oxygen atoms interacted with hydrogen located on aromatic rings of organic linkers. No major distortion in UiO-66 was observed.

A more thorough analysis was conducted using the independent gradient model based on Hirshfeld partition (IGMH) and atoms in molecule (AIM). IGMH–AIM is a computational scheme that encompasses information about the relative contributions of each atom to the electron density and can be used to evaluate the strength of intermolecular interactions within the context of adsorption. Anions, ferrocenium, and UiO-66 nodes were treated as distinct fragments, and interfragment interactions were quantified using the δg^inter^ index. The results of our analysis are visualized in Figure 4e, where blue, green, and red ellipsoids represent attractive interactions, weak van der Waals (vdW), and repulsive interactions, respectively.^46,47^ Chloride complexes exhibited attractive interactions between chloride and iron or zirconium (with a minor vdW interaction with linker hydrogen). In contrast, nitrate complexes displayed spatially distributed interaction regions with prominent and equivalent contributions from the constituent oxygen atoms. While chloride and nitrate are both monovalent, nitrate benefits from an overall larger interaction region due to each oxygen atom possessing a partial negative charge. This was more apparently manifested in nitrate–UiO-66 complexes, where a single constituent oxygen coordinated with zirconium, while two other oxygen atoms formed strong attractive interactions with the hydrogen on organic linkers. The geometric arrangement of oxygen constituents also likely has a non-negligible effect on bond formation and the value of sign(λ_2_)ρ. In octahedral anions such as sulfate and phosphate, a single constituent oxygen atom coordinated with either iron or zirconium, while other oxygen atoms engaged attractively with hydrogen from cyclopentadienyl rings or organic linkers. The nature of hydrogen interactions according to the values of sign(λ_2_)ρ resembled vdW attraction when interacting with cyclopentadienyl hydrogen and hydrogen bonding when interacting with organic linker hydrogen. Since phosphate is more negatively charged, interaction regions were coded with a deeper shade of blue, corresponding to more attractive interactions. The strength of attractive interactions followed the trend PO_4_^3–^ > SO_4_^2–^ > NO_3_^–^ > Cl^–^.

Enthalpies of adsorption (*H*_ads_) and free energies of adsorption (*G*_ads_) were calculated and are plotted in Figure 4f. Based on the results, all anion adsorption configurations were thermodynamically favored with adsorption following PO_4_^3–^ > SO_4_^2–^ > NO_3_^–^ > Cl^–^. Notably, PO_4_^3–^ exhibited an adsorption energy approximately twice as much as that of SO_4_^2–^ in a ferrocenium associated system and nearly three times as much in a UiO-66 associated system. This finding is particularly significant, since the density of UiO-66 nodes is far higher than that of ferrocenium pendants, meaning an elevated preference for PO_4_^3–^. The results obtained are in excellent agreement with our experiments and clearly manifested in multi-ion solution mixtures. Another aspect of the adsorption trend is that anions ranking higher in the list are less likely displaced by lower-ranked anions since they are able to form more stable complexes with the adsorbent.

## Conclusion

In this work, we demonstrated an application of MOFs for environmental remediation, specifically for the removal of oxyanion contaminants. UiO-66 had been functionalized with ferrocene and was employed as an anode in an electrochemical cell. Redox-mediated anion adsorption was achieved through electrochemical oxidation of the ferrocene pendants in Fc-UiO-66, which resulted in the migration–diffusion of anions into the pores. Oxidation potential was varied from 0.4 to 1.0 V, and preferential removal of anions followed a trend of PO_4_^3–^ > SO_4_^2–^ > NO_3_^–^ > Cl^–^ (10-fold concentration). Surprisingly, structural and electrochemical characterization of Fc-UiO-66 showed minimal evidence of steric hindrance at play. Instead, electronic structure calculations revealed that the oxyanion selectivity originated from a spatial distribution of charges due to oxygen constituents. Each oxygen constituent possessed a partial negative charge, which contributed to a larger interaction zone with the positively charged pore interior composed of ferrocenium pendants and zirconium nodes. Through IGMH–AIM analysis, we determined that attractive interactions present in oxyanion–UiO-66 complexes were stronger and more stable than those of oxyanion–ferrocenium, thereby being the most likely adsorbate–adsorbent configuration. Broadly speaking, our study is a demonstration of how molecular charge distribution in tandem with the local environment can result in the formation of highly specific complexes, similar to how biological ion channels function.^48^ Other MOF materials can also exploit this strategy to achieve the precise separation of small molecules for a wider range of applications.

## Methods

### Synthesis of UiO-66

UiO-66 was synthesized according to literature.^24,49^ A metal precursor solution was first prepared by depositing 125 mg of ZrCl_4_ into a glass vial containing 5 mL of DMF followed by the addition of 1 mL of 37% (w/w) HCl. The ligand solution was separately prepared by depositing 123 mg of terephthalic acid (H_2_BDC) in 10 mL of DMF. Both solutions were sonicated for at least 30 min before the ligand solution was dropwise added to the metal precursor solution. The resulting mixture was then placed in an oven at 80 °C for 12 h. After the mixture had cooled, it was then washed with DMF and acetone before drying in an oven at 60 °C. UiO-66 was activated by heating it in a vacuum oven at 120 °C for at least 48 h before any characterization was carried out.

### Synthesis of Fc-UiO-66

Ferrocene was incorporated using the reported solvent-assisted ligand incorporation (SALI) process^24^ in small batches. 48 mg of ferrocenecarboxylic acid was first dissolved in 3 mL of DMF before 32 mg of activated UiO-66 was added. The pale, chalky mixture was sonicated for 30 min and placed in an oil bath set to 60 °C for 24 h. Finally, a burgundy-colored mixture was obtained and washed with DMF and acetone before drying in an oven. Fc-UiO-66 was activated similarly, following the steps outlined for UiO-66.

### Material Characterization

Scanning electron micrographs were taken using a field emission scanning electron microscope (FE-SEM, JEOL JSM-7600F), while transmission electron micrographs were obtained with a transmission electron microscope (TEM, FEI Talos F200X) equipped with an energy-dispersive X-ray spectrometer (EDS). Electron paramagnetic resonance (EPR) spectroscopy was performed using a Bruker EMXplus-6/1 spectrometer with a 100 kHz modulation frequency.

Structural properties were investigated via powder X-ray diffraction spectroscopy (PXRD) on desolvated MOF samples in Bragg–Brentano geometry using a Bruker D8 Advance diffractometer equipped with a Ni filtered Cu Kα radiation (λ = 1.5406 Å, 40 kV, and 40 mA) source and a 1D LynxEye detector. Diffraction patterns were collected over a 2θ range of 5–80° at increments of 0.02°. Rietveld refinement of the full spectral range was performed using Profex^50^ (Ver. 5.2.4) with instrument specific parameters (see Note S1).

X-ray absorption near edge structure (XANES) and extended X-ray absorption fine structure (EXAFS) spectra were obtained in transmission mode using Si(111) crystal monochromators at the BL11B beamline at the Shanghai Synchrotron Radiation Facility (SSRF, Shanghai, China). A four-channel silicon drift detector (SDD) Bruker 5040 was used to record the spectra at room temperature. All samples were prepared by first compressing the materials into thin sheets of ∼1 cm and sealing with Kapton tape. Spectral data were subsequently processed and analyzed using Athena and Artemis from the Demeter software package.^51^

X-ray photoelectron spectroscopy (XPS) was performed using Thermo Fisher Scientific’s ESCALAB 250Xi XPS Microprobe system equipped with a monochromatic Al Kα X-ray source (*hv* = 1486.6 eV) operated at 150 W. Full spectrum scans were obtained using a pass energy of 100 eV with a step size of 1 eV, while high-resolution narrow scans were obtained using a pass energy of 20 eV with a step size of 0.1 eV. Charge correction was performed based on the adventitious C 1s carbon peak found at 284.8 eV. CasaXPS software (version 2.3.19) was used to process and analyze the obtained spectra. A Shirley background subtraction was first performed across the region of interest before component peaks were subsequently fitted with Gaussian–Lorentzian lines of 70:30 distribution.

The Brunauer–Emmett–Teller (BET) theory was used to estimate the specific surface area, and nitrogen adsorption–desorption isotherms were measured at 77 K using an Autosorb-iQ-MP-XR system (Quantachrome) after degassing at 150 °C for 6 h. The pore size distribution was subsequently determined using quenched solid state functional theory (QSDFT) provided by the proprietary ASWin (Quantachrome) software. The amount of ferrocene was determined using an inductively coupled plasma optical emission (ICP-OES) spectrometer (ICPE-9820, Shimadzu) after samples were dissolved in HNO_3_. Thermogravimetric analysis (TGA) was used to determine the number of missing organic linkers using a TA Q50 analyzer at a ramp rate of 5 °C min^–1^ from 30 to 900 °C under a continuous flow of dry, compressed air. Ion chromatography (IC) was conducted using Metrohm’s 930 Compact IC Flex to quantify the amount of anions removed. The IC system was equipped with a chemical suppressor module, and anions were separated using the Metrosep A Supp 16-100/4.0 analytical column with an attached guard column. IC data processing was performed using Metrohm’s MagIC Net 3.2 software.

### Electrode Fabrication and Electrochemical Characterization

Electrodes were fabricated by using a typical slurry and coating method. A slurry was first prepared with Fc-UiO-66, polyvinylidene fluoride (PVDF, *M*_w_ ∼ 180 000), and multiwalled carbon nanotubes (MWCNTs) in a ratio of 8:1:1 with *N*-methyl-2-pyrrolidone (NMP, 99.5%) as the solvent. After it was mixed thoroughly, the slurry was coated onto carbon paper (Shanghai Hesen Electronics Co. Ltd.) and dried at 60 °C overnight. Electrodes used in redox-mediated adsorption experiments were circular discs of area ∼5.5 cm^2^, while electrodes used in electrochemical characterization occupied square areas of 1 cm^2^.

Cyclic voltammetry (CV) and chronoamperometry experiments were performed using a standard 3-electrode setup consisting of a working electrode (1 × 1 cm^2^), Pt counter electrode (1.5 × 1.5 cm^2^), and saturated calomel (SCE) reference electrode. Ar gas was bubbled through the cell before experiments began and bubbled continuously while experiments were running. Measurements were taken after electrodes were conditioned in the relevant salt solutions overnight and cycled through 10 CV scans. For chronoamperometry experiments estimating the percentage of electroactive ferrocene, electrodes were cycled, and a step potential of −0.2 V was applied for 15 min to completely reduce ferrocenium.

### Redox-Mediated Adsorption of Anions

A homemade flow-by cell was constructed and assembled according to Figure S7. Fc-UiO-66-coated carbon paper served as the anode, while an activated carbon-coated carbon paper with a cation-exchange membrane (CEM, IONSEP) served as the cathode. The two electrodes were separated by an acrylic separator, which also served as the flow channel. A perforated nylon mesh was also placed within the channel to ensure flow uniformity.

A batch-mode operation was adopted, and a peristaltic pump (BT100S, Leadfluid) was used to cycle solutions through the cell while a programmable sourcemeter (SMU 2450, Keithley) was used to oxidize/reduce the electrode. The salt solutions were cycled at a constant flow of 30 mL min^–1^ from a reservoir, and total volume was kept constant at 50 mL for all experiments. To negate the influence of dissolved oxygen, Ar gas was bubbled through the reservoir 10 min before the experiment began and flowed continuously while the experiment was running. Dilute amounts of either HCl or NaOH were used to regulate the pH in the pH-dependent experiments. A pH probe (ET042, eDAQ) was placed near the water outlet to record changes in the pH.

Ion adsorption was achieved by supplying a potential step between 0.4 and 1.0 V at the anode for 15 min followed by −0.2 V for 15 min to regenerate the electrodes. To quantify the amount of anion removed, aliquots of the effluent solution were taken before and after electrochemical oxidation for IC analysis. Aliquot sampling was performed after 3 redox cycles to prevent any spurious readings. The normalized amount of anion removed, η is given as
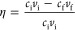
1where *c*_i_ and *c*_f_ are the effluent anion concentrations (mM) before and after oxidation, respectively. *v*_i_ and *v*_f_ are the volumes (L) before and after first aliquot sampling, respectively. The equation for ion selectivity of an oxyanion over Cl^–^ is given as
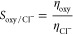
2

The apparent selectivity for a solution comprising *n* number of competitor ions is given as
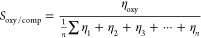
3where the denominator denotes an average, normalized value of competitor ions removed.

### Postadsorption *Ex Situ* XPS

*Ex situ* XPS was carried out on pieces of electrodes (∼1 cm^2^) cut from electrodes used in independent dual-ion adsorption experiments. Each electrode had been cycled at least 10 times before ending the run in a state of oxidation at the target potential. The reduction state was obtained after cycling at an oxidation potential of 0.8 V and ending at −0.2 V. Each electrode piece was washed with copious amounts of water to remove excess ions on the surface and dried at 100 °C overnight before the XPS experiments were performed.

### Computational Methods

Density functional theory (DFT) calculations were performed using Gaussian 16^52^ and utilized the B3LYP exchange correlation functional, complemented by D3 dispersion correction.^53^ Main group elements were treated using 6-31G(d) calculation basis sets, whereas metal elements employed pseudopotential basis sets based on solvation model density (SMD).^54^ UiO-66 structure was modeled after two specific units based on literature^55^ and represented using a resource efficient, finite monomer structure.^56^

Visual representations of electrostatic potential (ESP), highest occupied molecular orbitals (HOMO), and lowest unoccupied molecular orbitals (LUMO) along with an independent gradient model based on Hirshfeld partition (IGMH) and atoms in molecule (AIM) analyses were achieved using Multiwfn calculations in conjunction with the visual molecular dynamics (VMD) software. Multiwfn was also used for localized orbital locator (LOL) analysis encompassing projection mapping in three distinct directions.^57^

## Supporting Information Available

Additional experimental characterization, flow-by cell schematic, DFT models, Rietveld refinement, EXAFS parameters, EDS, XPS, TGA, chronoamperometry, redox-mediated adsorption in dual-ion solution results (PDF)
