# Supplementary material for: Electrochemical Selective Removal of Oxyanions in a Ferrocene-Doped Metal–Organic Framework
Source: ACS Nano. 2024 Oct 14;18(42):29067–77. doi: 10.1021/acsnano.4c10206 (PMC11581342; doi:10.1021/acsnano.4c10206)
Supplement: Supplementary file 1 — nn4c10206_si_001.pdf [file nn4c10206_si_001.pdf]

Supporting Information

Electrochemical Selective Removal of Oxyanions in a Ferrocene-doped Metal-Organic Framework

Zhi Yi Leong[1], Jingjing Yao[1#], Niels Boon[2], Hüseyin Burak Eral[2], Dong-Sheng Li[3], Remco Hartkamp[2*], Hui Ying Yang[1*]

[1]Pillar of Engineering Product Development (EPD), Singapore University of Technology and Design, 8 Somapah Road, 487372, Singapore. E-mail: yanghuiying@sutd.edu.sg

[2]Process & Energy Department, Delft University of Technology, Leeghwaterstraat 39, Delft, 2628 CB The Netherlands. E-mail: r.m.hartkamp@tudelft.nl

[3]College of Materials and Chemical Engineering, Key Laboratory of Inorganic Nonmetallic Crystalline and Energy Conversion Materials, China Three Gorges University, Yichang 443002, China

# Contributed equally

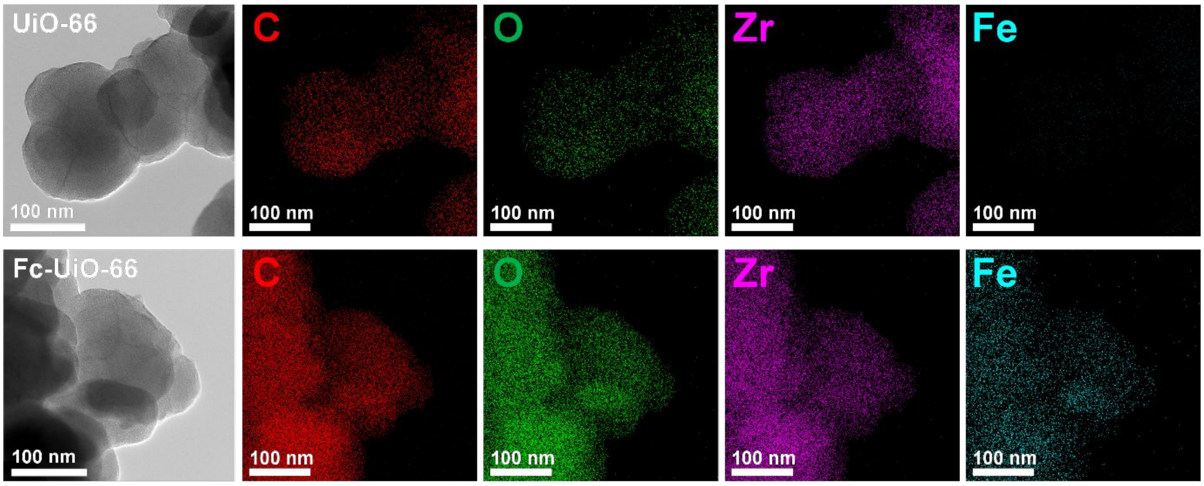

Fig. S1 EDS spectra of UiO-66 and Fc-UiO-66.

Supplementary Note S1

Rietveld refinement of powder X-ray diffraction (PXRD) data

PXRD measurements were performed on desolvated MOF samples after heating in vacuum at 180 °C for 24 h. Rietveld refinement was performed across the full 2θ range of 5 – 80° using an initial structure solution sourced from literature[1]. Minor displacements in atomic positions were allowed in accordance to physically acceptable values for bond distances. Zr cluster occupancy was fixed at 1 while occupancies of terminal μ_3–O and μ_3–OH groups were allowed to vary such that positive charge densities on the zirconium atoms were minimized. The occupancy of the organic linker was freely varied to simulate linker defects. Fig. S2 shows the final Rietveld refinement plot for UiO-66 and Table. S1 shows the refined parameters.

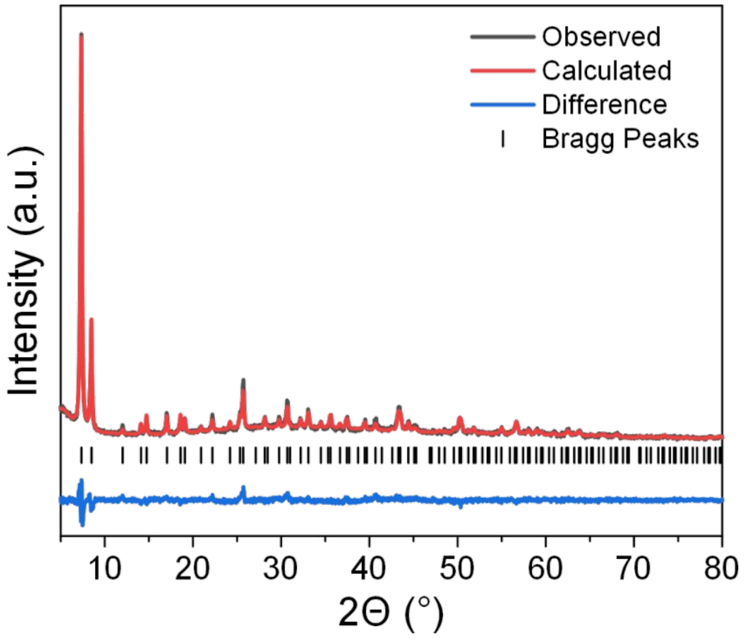

Fig. S2 Final Rietveld refinement plot of UiO-66. Black lines: Experimental pattern. Red lines: Calculated Rietveld pattern. Blue lines: Residual curve. Black ticks: Allowed reflections.

	UiO-66
Crystal system	Cubic
Space group	$Fm\bar{3}m$
Refined composition	$Zr_6C_{39.4}H_{23.3}O_{27.7}$
Unit cell parameters	
$a = b = c$ [Å]	20.7870
$\alpha = \beta = \gamma$ [°]	90
V = [Å^3]	8982.05
R_{wp} [%]	7.78
R_{exp} [%]	5.08
χ^2	2.35
GoF	1.53

Table. S1 Results of Rietveld refinement on UiO-66 PXRD data.

Supplementary Note S2

Extended X-ray absorption fine structure (EXAFS) fitting and analysis

Preliminary data normalization and background removal were performed in Athena before fitting of EXAFS data was performed in Artemis[2]. Using crystal data obtained from refinement as input for FEFF calculations, three single scattering paths were used for Zr scattering in UiO-66. These paths belonged to Zr-$O_{\mu 3}$ (terminal oxygen), Zr-O (carboxylate oxygen) and Zr-Zr (zirconium atom along octahedral edge of Zr cluster). The fitted Fourier transform of k^3-weighted EXAFS data is shown in Fig. S3.

X-ray absorption spectra at the Fe K-edge was obtained for pure ferrocene and Fc-UiO-66 samples. First shell fitting of the Fe K-edge for pure ferrocene was performed using a single Fe-C scattering path and a double scattering path contributed from two carbon atoms in the cyclopentadienyl ring. The plot of the fit is shown in Fig. S4. Compared to the EXAFS data of pure ferrocene, ferrocene in Fc-DU-01 showed drastically lower amplitudes across the entire R-space and more complex features originating from its distorted geometry. Only Fe-C single scattering paths were considered for the fit and spectral features do not suggest any influence from Zr atoms. Fitted parameters of pure ferrocene and Fc-DU-01 at the Fe K-edge are shown in Tables. S3 and S4 respectively.

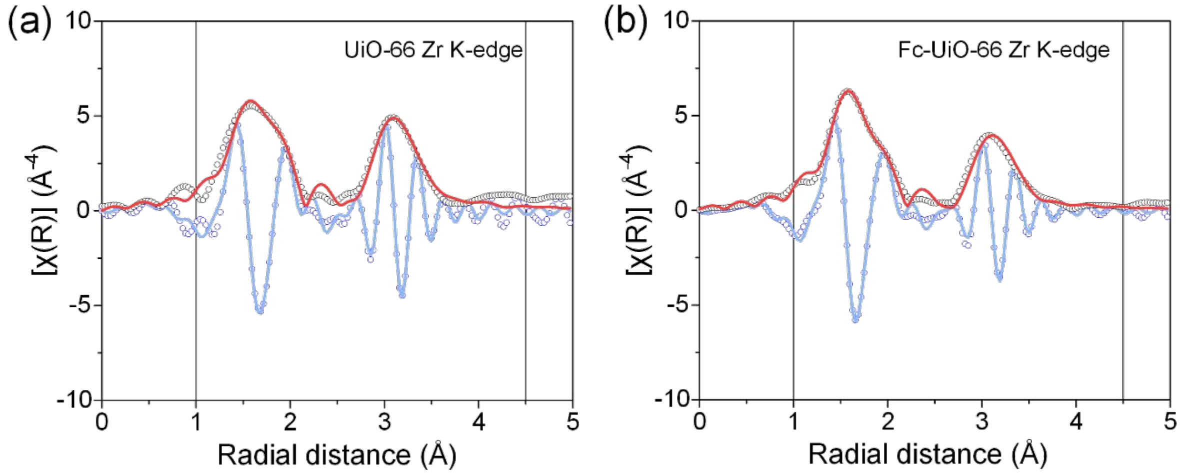

Fig. S3 Phase-uncorrected Fourier transform of k^3-weighted EXAFS data taken at the Zr K-edge for **(a)** UiO-66 and **(b)** Fc-UiO-66. Black circles: Raw modulus data. Red line: Fit to modulus. Blue circles: Raw imaginary component. Sky blue line: Fit to imaginary component. Black vertical lines: Fitting window.

	UiO-66	Fc-UiO-66
R-factor	0.017	0.008
ΔE_0 [eV]	3.307 ± 0.910	3.300 ± 0.626
S_0^2	1.017 ± 0.078	1.017
R(Zr-O$_{\mu3}$) [Å]	2.132 ± 0.010	2.140 ± 0.006
σ^2(Zr-O$_{\mu3}$) [Å^2]	0.0032 ± 0.0015	0.0027 ± 0.0005
R(Zr-O) [Å]	2.277 ± 0.010	2.291 ± 0.007
σ^2(Zr-O) [Å^2]	0.0025 ± 0.0016	0.0013 ± 0.0006
R(Zr-Zr) [Å]	3.590 ± 0.007	3.537 ± 0.006
σ^2(Zr-Zr) [Å^2]	0.0058 ± 0.0006	0.0075 ± 0.0005

Table. S2 EXAFS fitting parameters for Zr K-edge.

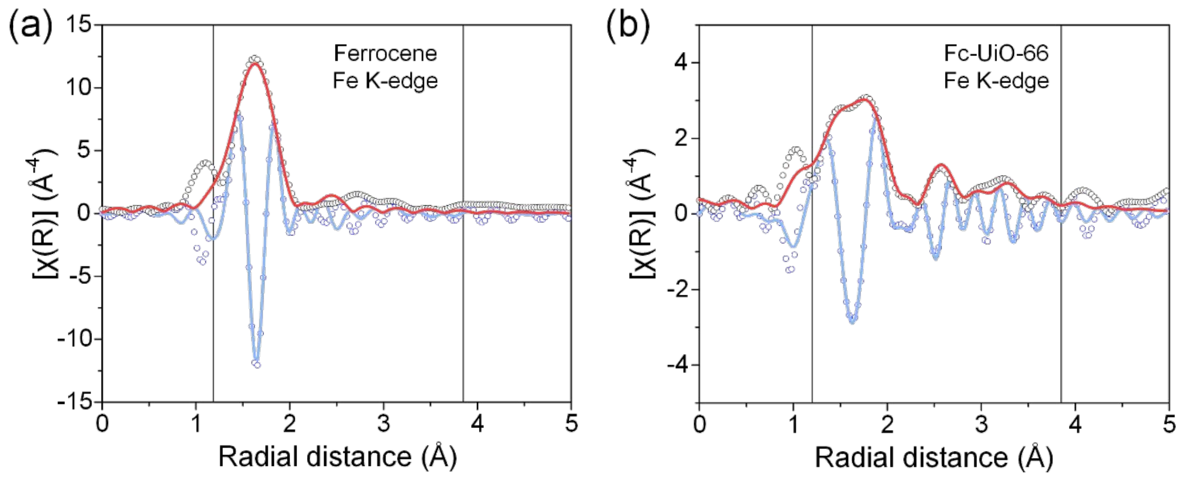

Fig. S4 Phase-uncorrected Fourier transform of k^3-weighted EXAFS data taken at the Fe K-edge for **(a)** ferrocene and **(b)** Fc-UiO-66. Black circles: Raw modulus data. Red line: Fit to modulus. Blue circles: Raw imaginary component. Sky blue line: Fit to imaginary component. Black vertical lines: Fitting window.

	Pure ferrocene
R-factor	0.020
ΔE_0 [eV]	0.055 ± 0.447
S_0^2	0.991 ± 0.370
R(Fe-C) [Å]	2.063 ± 0.024
σ^2(Fe-C) [Å^2]	0.0040 ± 0.0031
R(Fe-C_1-C_2) [Å]	2.765 ± 0.073
σ^2(Fe-C_1-C_2) [Å^2]	0.0078 ± 0.010

Table. S3 EXAFS fitting parameters for Fe K-edge for pure ferrocene.

	Fc-UiO-66
R-factor	0.016
ΔE_0 [eV]	0.416 ± 1.51
S_0^2	0.991
R(Fe-C_1) [Å]	2.007 ± 0.014
σ^2(Fe-C_1) [Å^2]	0.0017 ± 0.0009
R(Fe-C_2) [Å]	2.139 ± 0.013
σ^2(Fe-C_2) [Å^2]	0.0051 ± 0.0009
R(Fe-C_3) [Å]	2.570 ± 0.017
σ^2(Fe-C_3) [Å^2]	0.0006 ± 0.0014
R(Fe-C_4) [Å]	3.421 ± 0.045
σ^2(Fe-C_4) [Å^2]	0.0008 ± 0.0051

Table. S4 EXAFS fitting parameters for Fe K-edge for Fc-DU-01. C_1 to C_3 refer to carbon atoms within the cyclopentadienyl ring whereas C_4 denotes the carbon atom on the carboxyl group.

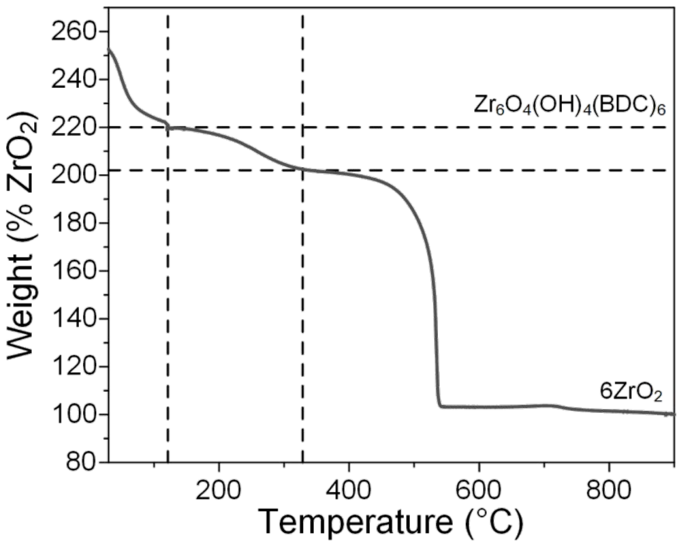

Fig. S5 TGA curve of pristine UiO-66 normalized to ZrO_2. An initial sharp drop in mass was attributed to loss of adsorbed water molecules on the surface of MOF crystallites whereas a second more gradual decrease in mass was attributed to solvent molecules trapped within the MOF. Assuming only loss of organic ligands during thermal decomposition, the difference in composition between as-synthesized UiO-66 and an ideal unit of UiO-66 is approximately 1.02 BDC linker.

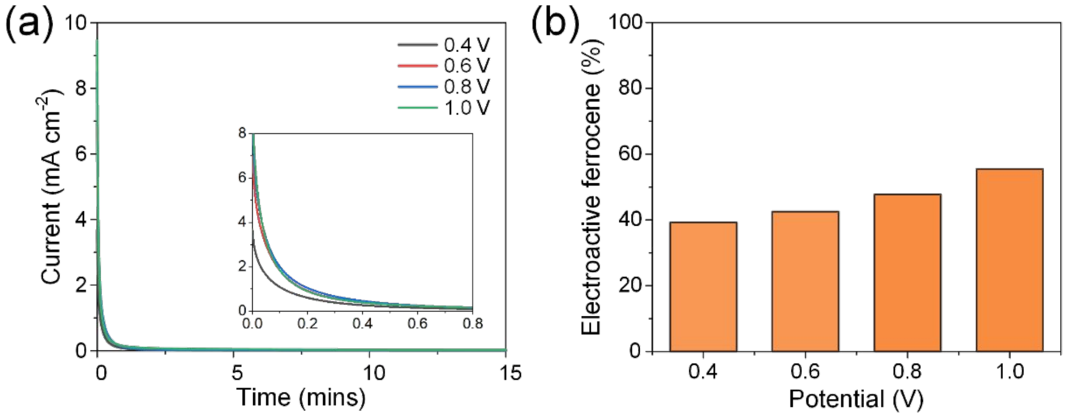

Fig. S6 (a) Representative chronoamperometry curves in 0.1 M NaCl solution subjected to different potentials. Insert shows close-up of curve at the beginning of the potential step. **(b)** Percentage of electroactive ferrocene.

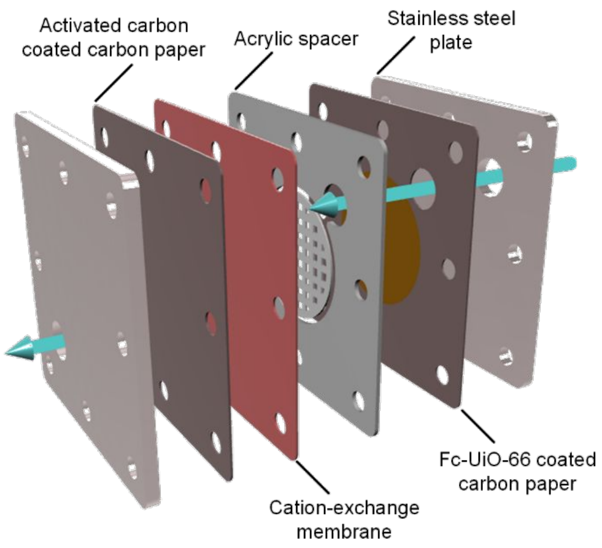

Fig. S7 Schematic depicting the assembly of an electrochemical flow-by cell for ion separation. Water flows (cyan arrows) through the inlet, traverses across the perforated, nylon mesh and exits through an oppositely positioned outlet.

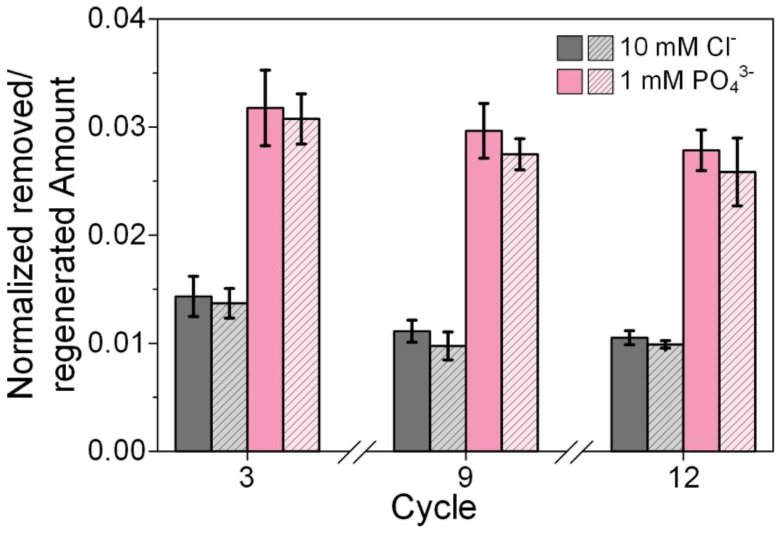

Fig. S8 Redox-mediated adsorption (solid columns) and regeneration (shaded columns) of 1 mM PO_4^{3-} with 10 mM Cl^- as background. Oxidation potential was 0.8 V.

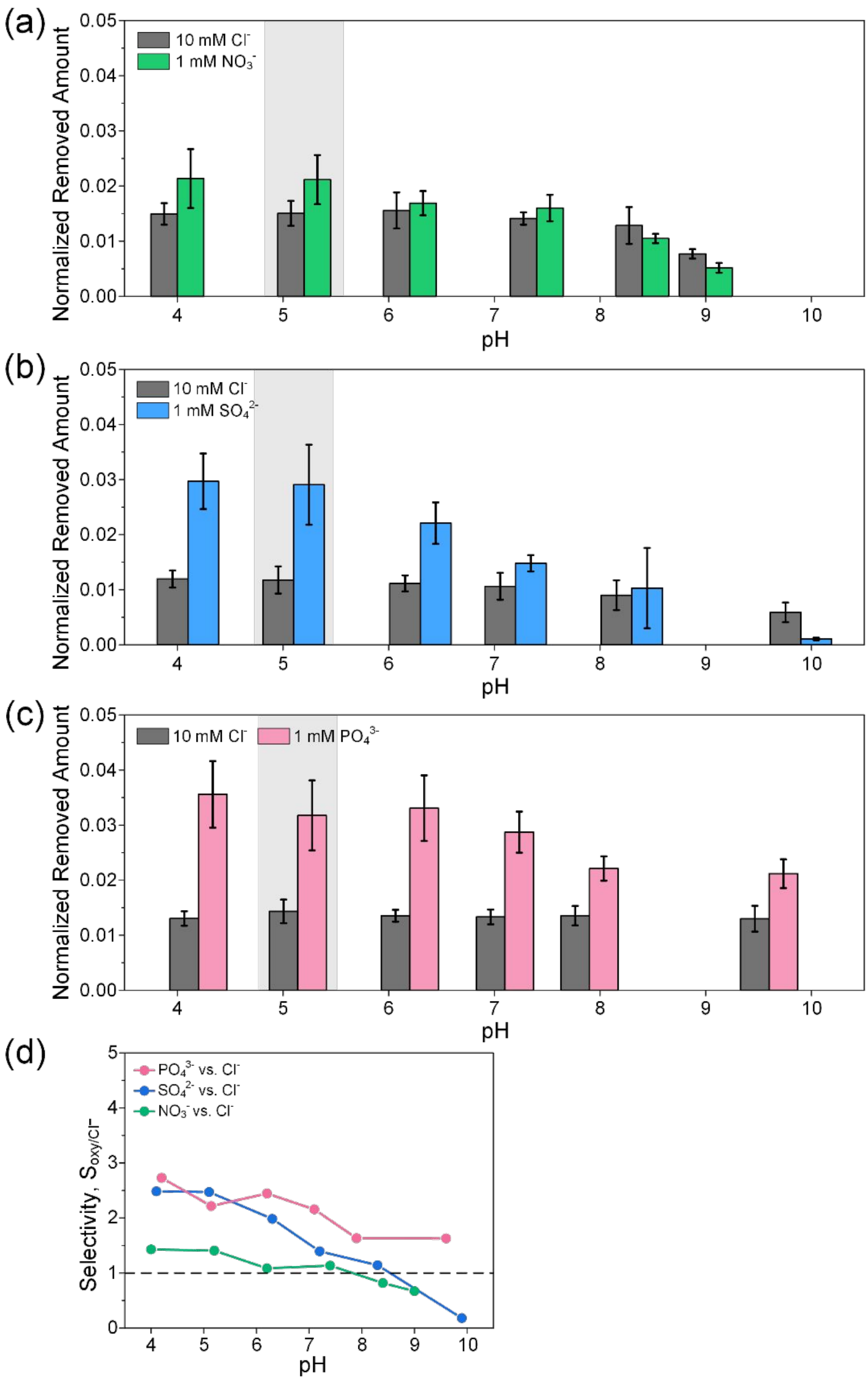

Fig. S9 Normalised redox-mediated adsorption of 1 mM **(a)** NO_3^-, **(b)** SO_4^{2-} and **(c)** PO_4^{3-} with 10 mM Cl^- as background under an oxidation potential of 0.8 V. Shaded region of column chart indicates that measurements were taken in the natural pH of the solution. **(d)** Ion selectivities of oxyanions calculated from (a – c). An ion-selectivity of 1 (dashed line) indicates no selectivity.

Supplementary Note S3

XPS spectra of as-synthesized UiO-66 and Fc-UiO-66 after desolvation are shown in Fig. S10. Zr 3d peaks of UiO-66 and its ferrocene-doped counterpart were remarkably similar, both showing typical deconvoluted peaks of Zr $3d_{3/2}$ and Zr $3d_{5/2}$ peaks at 185.3 and 185.2 eV, respectively. O 1s peak could be deconvoluted into three component peaks located at 533.0, 531.8 and 530.2 eV. Correspondingly, these peaks are assigned to O-C=O (533.0 eV), Zr-O-C (531.8 eV) and Zr-O-Zr (530.2 eV)[3, 4, 5]. An increase in relative intensity of the O-C=O peak was observed for Fc-UiO-66 and could be attributed to the additional carboxylate group contributed by ferrocenecarboxylic acid. Fe 2p peaks were observed in Fc-UiO-66 showing spin-orbit splitting components, Fe $2p_{1/2}$ and Fe $2p_{3/2}$ [6] separated by an energy difference of 12.7 eV. The peaks of Fe $2p_{1/2}$ and Fe $2p_{3/2}$ were comprised of substructures associated to Fe^{2+} (720.6 and 707.8 eV) and Fe^{3+} (724.8 and 711.9 eV)[7, 8, 9] states. Under normal circumstances, iron coordination in ferrocene is low spin and will unambiguously exhibit only Fe^{2+} peaks. However, due to a distortion in ferrocene geometry (i.e. bent ferrocene structure), charge distribution is no longer uniformly distributed across the cyclopentadienyl rings which leads to the presence of substructures corresponding to the Fe^{3+} state.

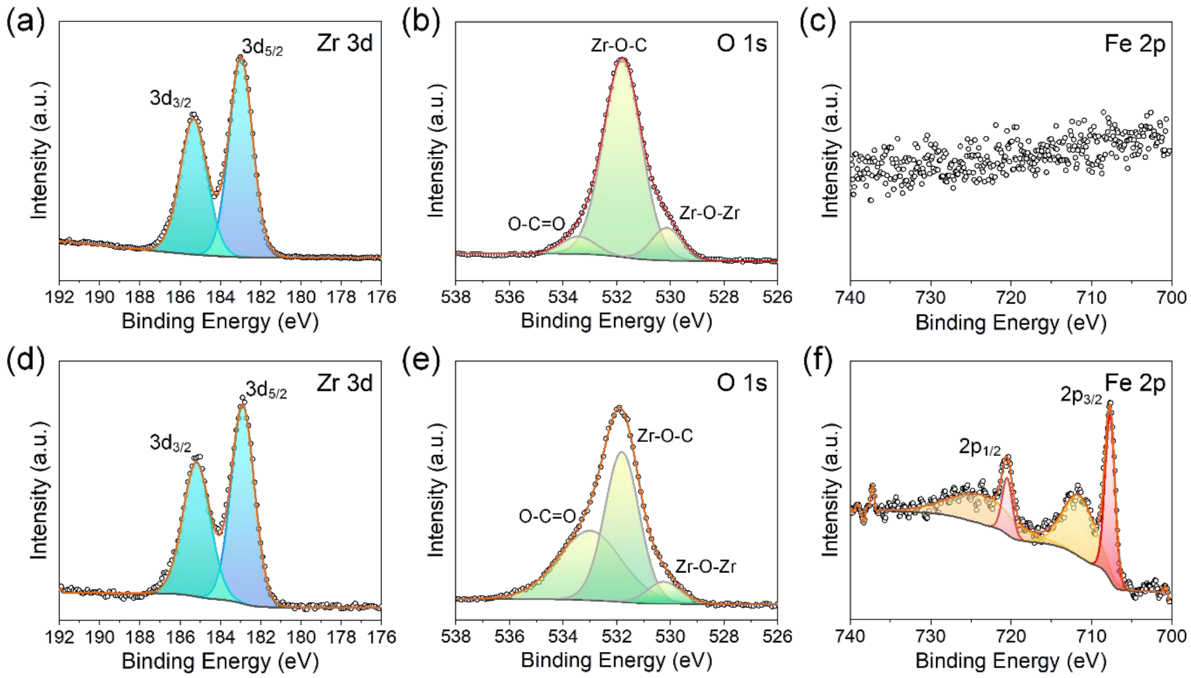

Fig. S10 High-resolution XPS spectra of **(a)** Zr 3d, **(b)** O 1s and **(c)** Fe 2p core regions of desolvated UiO-66 along with **(d)** Zr 3d, **(e)** O 1s and **(f)** Fe 2p core regions of desolvated Fc-UiO-66.

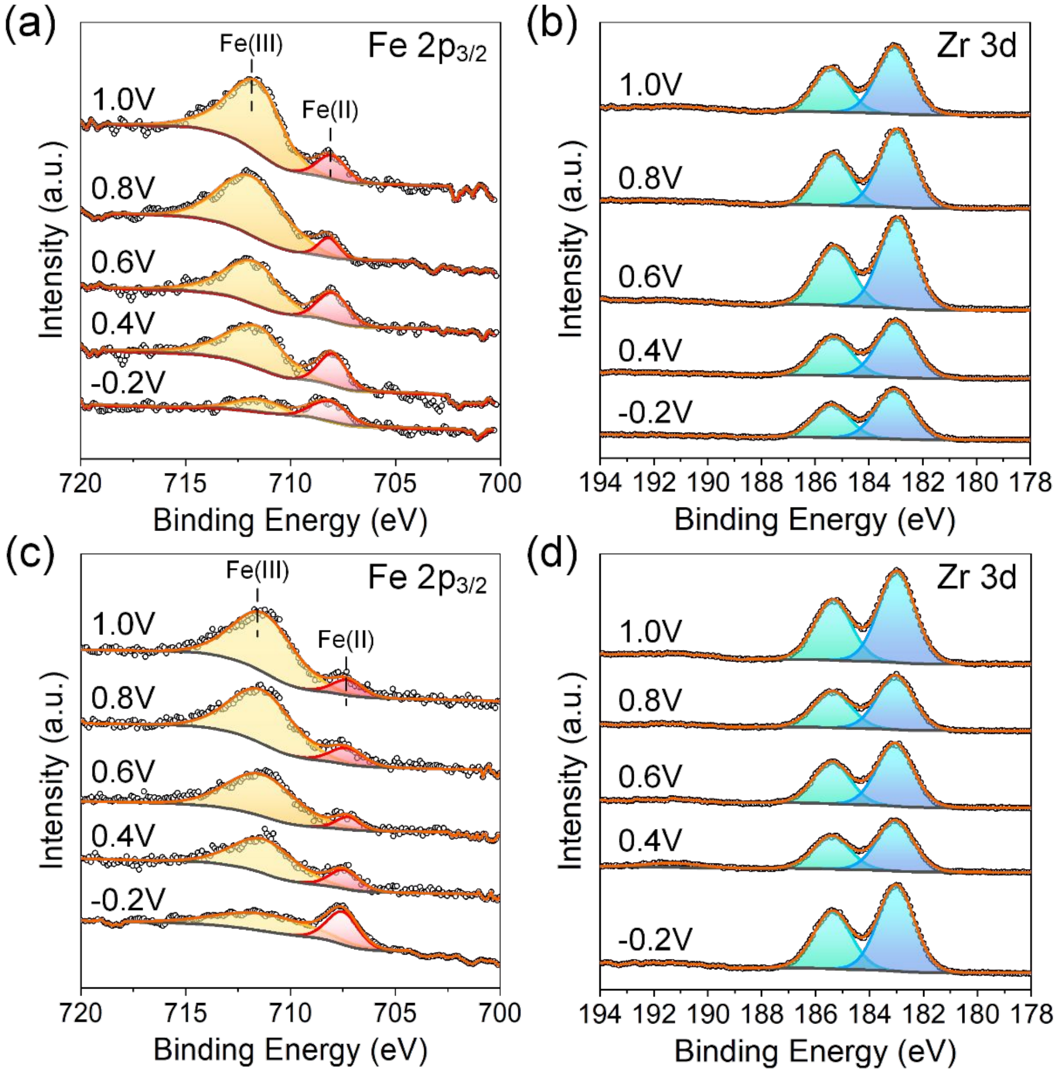

Fig. S11 High-resolution *ex-situ* XPS spectra of electrochemically oxidised Fc-UiO-66 anode in **(a, b)** 1 mM $NaNO_3$ and 10 mM NaCl solution; **(c, d)** 1 mM Na_2SO_4 and 10 mM NaCl solution mixture.

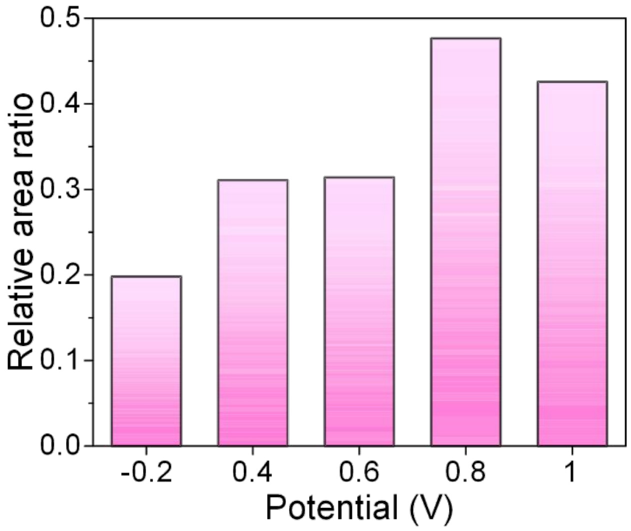

Fig. S12 Relative area ratio of the broad feature centred at 191.4 eV to Zr $3d_{5/2}$ in Zr 3d XPS spectra. XPS was taken when the electrode was oxidised in 1 mM NaH_2PO_4 and 10 mM NaCl solution mixture.

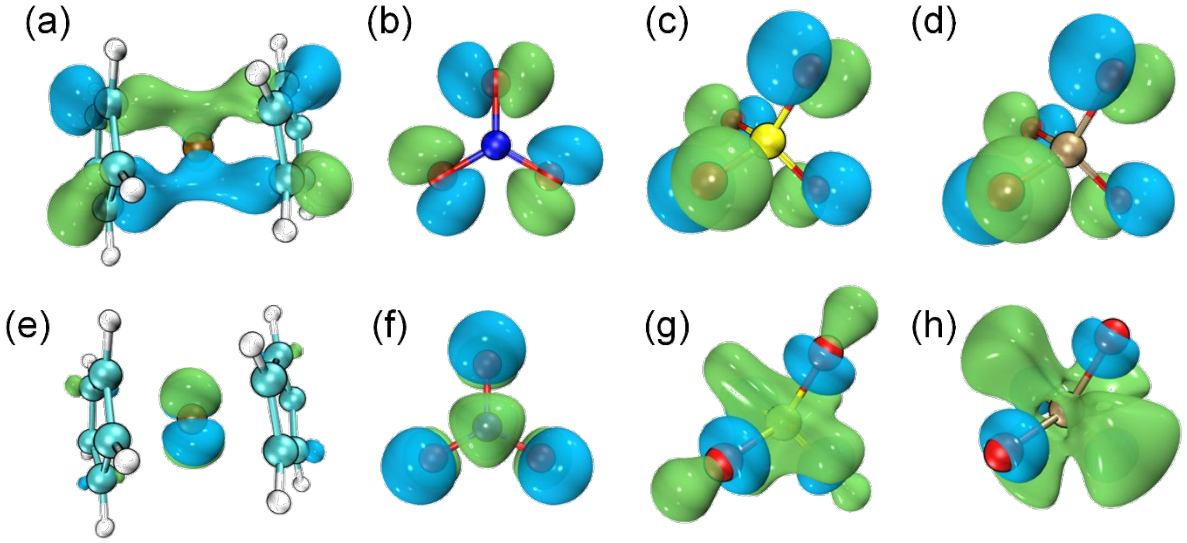

Fig. S13 HOMO of **(a)** ferrocene, **(b)** NO_3^-, **(c)** SO_4^{2-} and **(d)** PO_4^{3-}. LUMO of **(e)** ferrocenium, **(f)** NO_3^-, **(g)** SO_4^{2-} and **(h)** PO_4^{3-}.

	HOMO (a.u.)	LUMO (a.u.)	E_{gap} (a.u.)
Ferrocenium	-0.12863	-0.0063	0.1223
Cl^-	0.01479	0.64337	0.6286
NO_3^-	0.00039	0.21679	0.2164
SO_4^{2-}	0.19542	0.56173	0.3663
PO_4^{3-}	0.44805	0.77615	0.3281

Table. S5 HOMO, LUMO and E_{gap} for ferrocenium, Cl^-, NO_3^- , SO_4^{2-} and PO_4^{3-}.

	Farthest distance between atoms (Å)	Van der Waals Radius of O atom (Å)	Molecular radius (Å)
Cl^-	-	-	1.81
NO_3^-	2.19	1.52	2.62
SO_4^{2-}	2.49	1.52	2.77
PO_4^{3-}	2.61	1.52	2.83

Table. S6 Calculated physical parameters of Cl^-, NO_3^- , SO_4^{2-} and PO_4^{3-}.

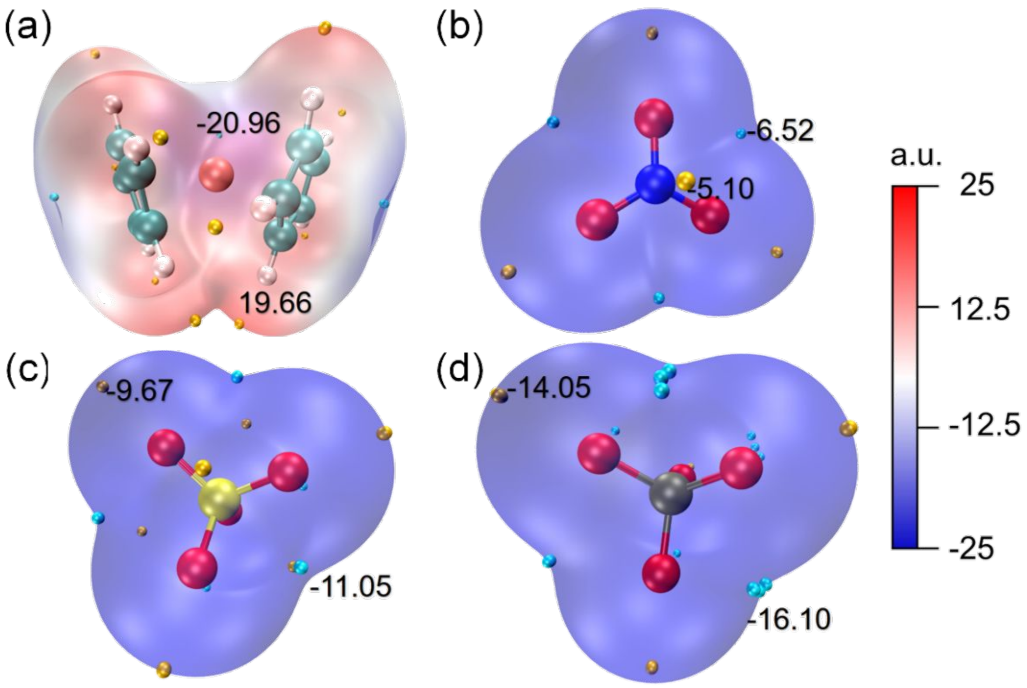

Fig. S14 ESP of **(a)** ferrocenium, **(b)** NO_3^-, **(c)** SO_4^{2-} and **(d)** PO_4^{3-}. Yellow and blue dots on the molecular surface indicate likely adsorption sites based on regions of high and low electrostatic potential, respectively.

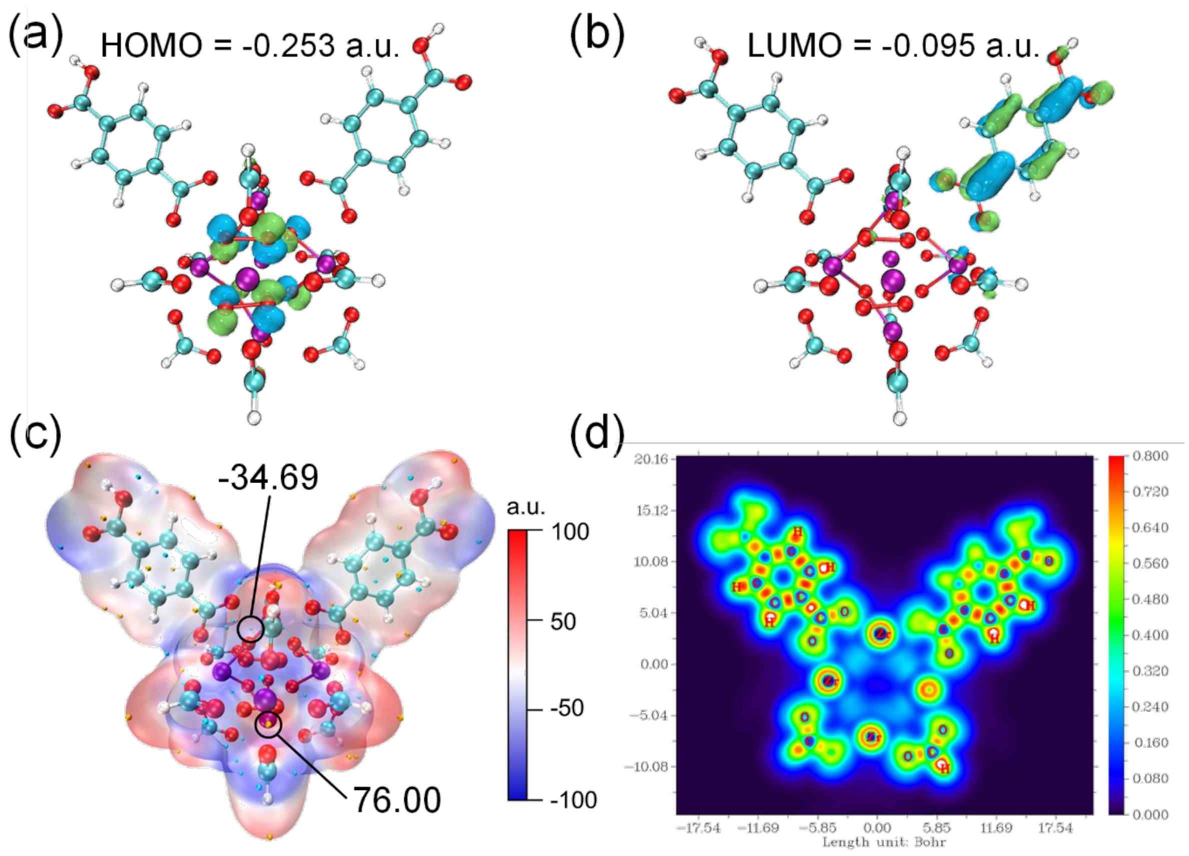

Fig. S15 (a) HOMO, **(b)** LUMO, **(c)** ESP and **(d)** LOL mapping of UiO-66.

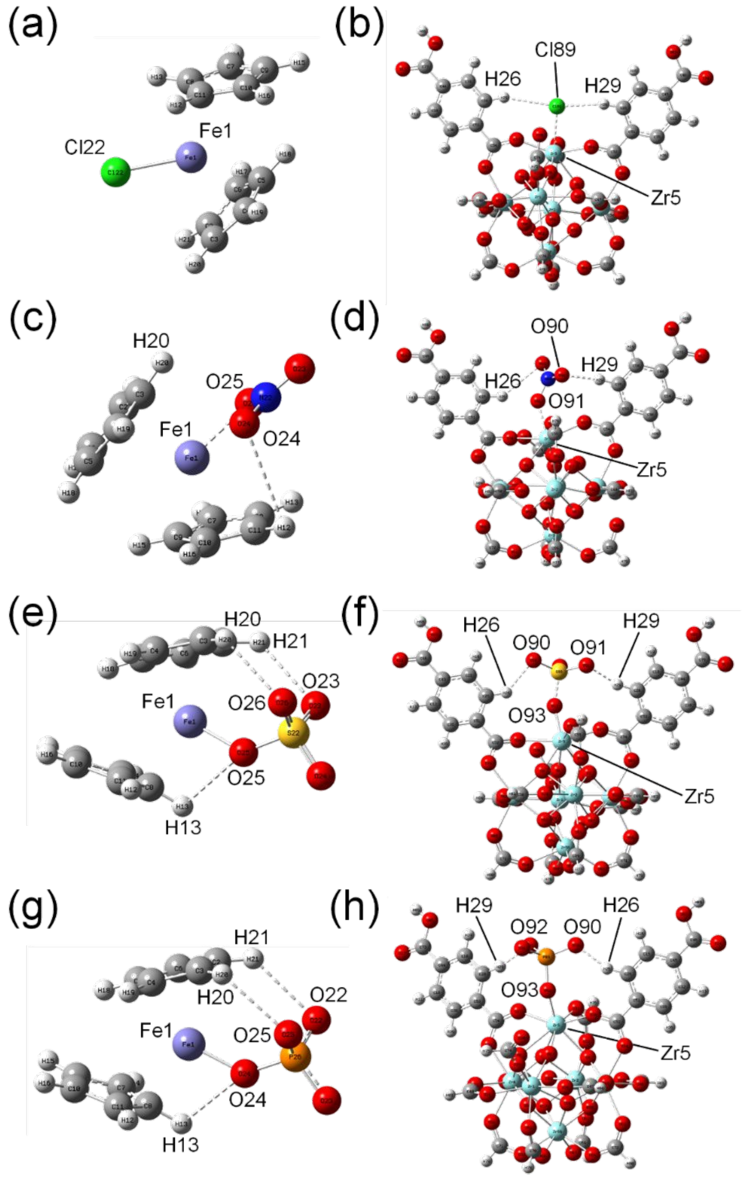

Fig. S16 Optimal adsorption configurations of **(a)** Cl^-, **(c)** NO_3^-, **(e)** SO_4^{2-} and **(g)** PO_4^{3-} with ferrocene. Correspondingly, optimal adsorption configurations of **(b)** Cl^-, **(d)** NO_3^-, **(f)** SO_4^{2-} and **(h)** PO_4^{3-} with UiO-66.

System	Bond type	Bond length (Å)
Ferrocenium & Cl^-	Fe1-Cl22	2.318
Ferrocenium & NO_3^-	Fe1-O24	2.244
	Fe1-O25	2.245
	H20-O24	2.893
	H20-O25	2.891
Ferrocenium & SO_4^{2-}	Fe1-O25	1.883
	H20-O26	2.516
	H21-O23	2.54
	H13-O25	2.747
Ferrocenium & PO_4^{3-}	Fe1-O24	1.857
	H13-O24	2.75
	H20-O25	2.567
	H21-O22	2.536

Table. S7 Bond type and length in ferrocenium adsorption environment.

System	Bond type	Bond length (Å)
UiO-66 & Cl^-	H29-Cl89	2.966
	H26-Cl89	2.678
	Zr5-Cl89	2.619
UiO-66 & NO_3^-	H29-O90	2.212
	H26-O91	2.358
	Zr5-O91	2.16
UiO-66 & SO_4^{2-}	H29-O91	2.048
	H26-O90	2.246
	Zr5-O93	1.965
UiO-66 & PO_4^{3-}	H29-O92	2.107
	H26-O90	2.028
	Zr5-O93	1.996

Table. S8 Bond type and length in UiO-66 adsorption environment.

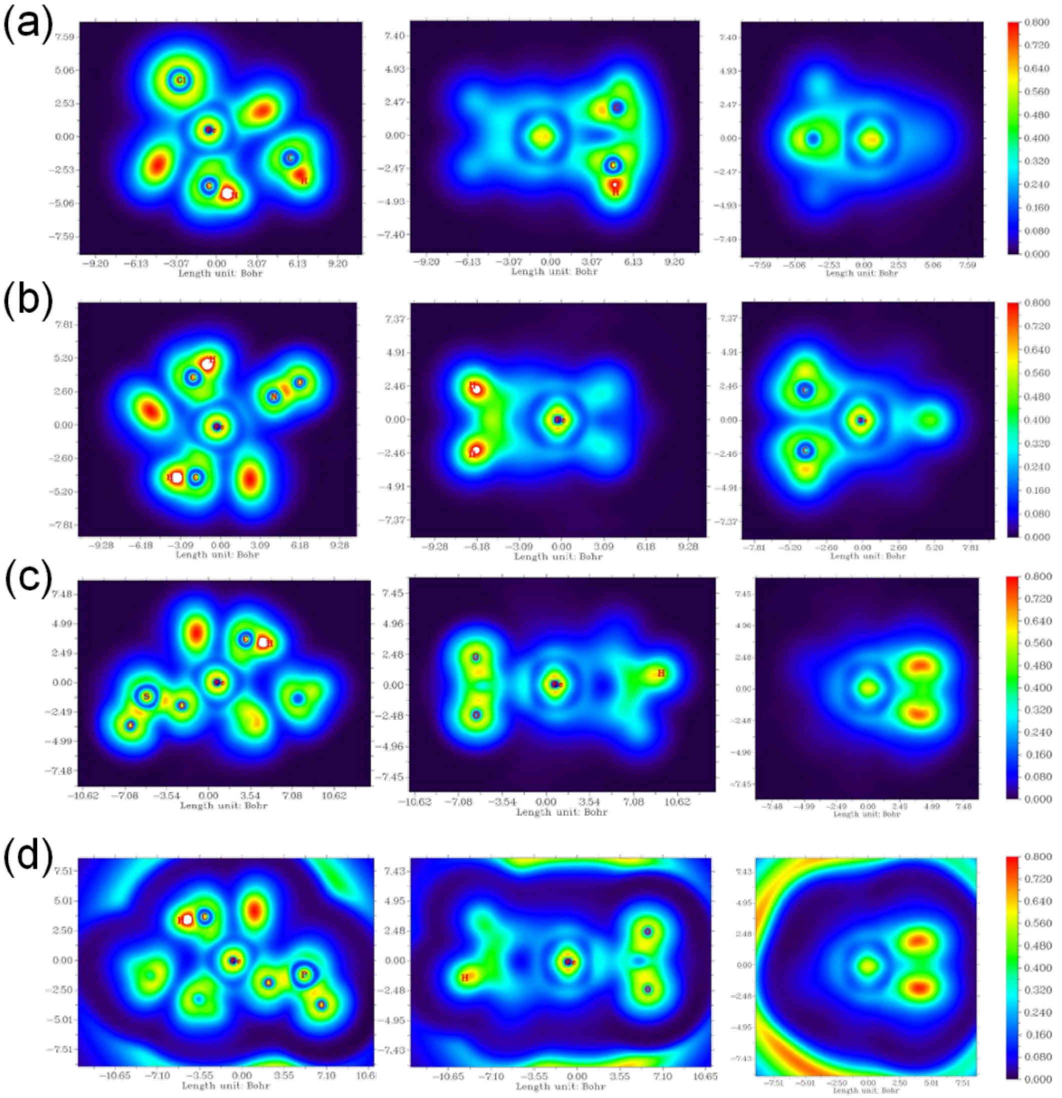

Fig. S17 LOL mapping of **(a)** Cl^-, **(b)** NO_3^-, **(c)** SO_4^{2-} and **(d)** PO_4^{3-} on ferrocene. First column: XY direction. Second column: XZ direction. Third column: YZ direction.

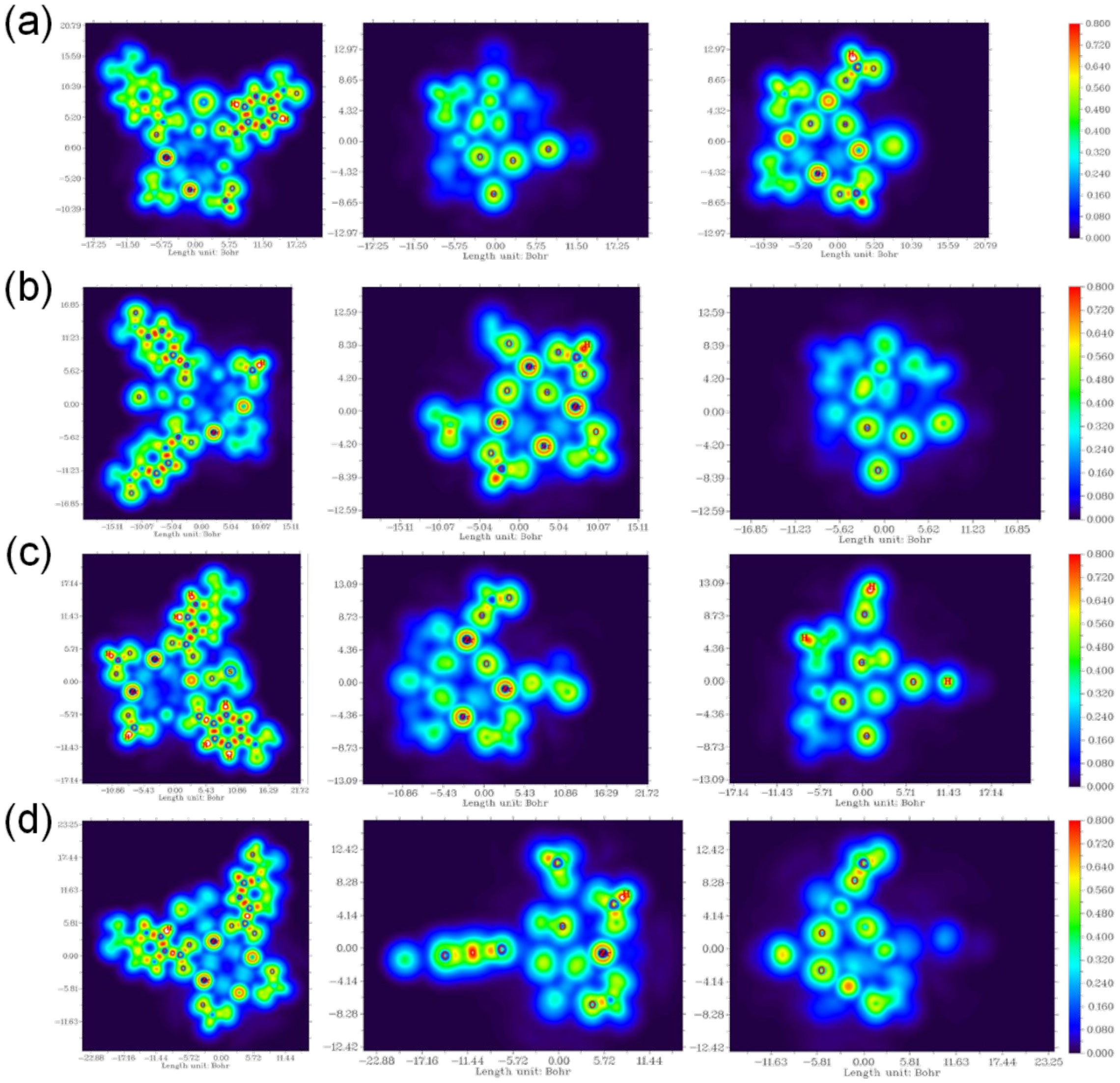

Fig. S18 LOL mapping of **(a)** Cl^-, **(b)** NO_3^-, **(c)** SO_4^{2-} and **(d)** PO_4^{3-} on UiO-66. First column: XY direction. Second column: XZ direction. Third column: YZ direction.

References

1. Lee S, Bürgi H-B, Alshmimri SA, Yaghi OM. Impact of disordered guest–framework interactions on the crystallography of metal–organic frameworks. *Journal of the American Chemical Society* 140, 8958-8964 (2018).

2. Ravel B, Newville M. ATHENA, ARTEMIS, HEPHAESTUS: data analysis for X-ray absorption spectroscopy using IFEFFIT. *Journal of synchrotron radiation* 12, 537-541 (2005).

3. Mukhopadhyay S, Shimoni R, Liberman I, Ifraemov R, Rozenberg I, Hod I. Assembly of a Metal–Organic Framework (MOF) Membrane on a Solid Electrocatalyst: Introducing Molecular-Level Control Over Heterogeneous CO2 Reduction. *Angewandte Chemie International Edition* 60, 13423-13429 (2021).

4. Wang Y*, et al.* Missing-node directed synthesis of hierarchical pores on a zirconium metal–organic framework with tunable porosity and enhanced surface acidity via a microdroplet flow reaction. *Journal of Materials Chemistry A* 5, 22372-22379 (2017).

5. Paz R*, et al.* Zirconium-organic framework as a novel adsorbent for arsenate remediation from aqueous solutions. *Journal of Molecular Liquids* 356, 118957 (2022).

6. Dong H*, et al.* Depletable peroxidase-like activity of Fe3O4 nanozymes accompanied with separate migration of electrons and iron ions. *Nature Communications* 13, 5365 (2022).

7. Gerber SJ, Erasmus E. Electronic effects of metal hexacyanoferrates: An XPS and FTIR study. *Materials Chemistry and Physics* 203, 73-81 (2018).

8. Liang B*, et al.* Facile fabrication of bimetallic Fe 2 P–Ni 2 P heterostructure for boosted oxygen evolution. *Journal of Materials Science: Materials in Electronics* 32, 23420-23428 (2021).

9. Grosvenor A, Kobe B, Biesinger MC, McIntyre N. Investigation of multiplet splitting of Fe 2p XPS spectra and bonding in iron compounds. *Surface and Interface Analysis: An International Journal devoted to the development and application of techniques for the analysis of surfaces, interfaces and thin films* 36, 1564-1574 (2004).
